# Polar mutagenesis of polycistronic bacterial transcriptional units using Cas12a

**DOI:** 10.1186/s12934-022-01844-y

**Published:** 2022-07-13

**Authors:** Antoine Graffeuil, Julio Guerrero-Castro, Aster Assefa, Bernt Eric Uhlin, David A. Cisneros

**Affiliations:** 1 Department of Molecular Biology, Umeå University, Umeå, Sweden; 2 The Laboratory for Molecular Infection Medicine Sweden (MIMS), Umeå University, Umeå, Sweden; 3 Umeå Centre for Microbial Research (UCMR), Umeå University, Umeå, Sweden

**Keywords:** Cas12a, CRISPR mutagenesis, Polycistronic operons, Intracellular ATP, Markerless genome editing

## Abstract

**Background:**

Functionally related genes in bacteria are often organized and transcribed as polycistronic transcriptional units. Examples are the *fim* operon, which codes for biogenesis of type 1 fimbriae in *Escherichia coli,* and the *atp* operon, which codes for the FoF1 ATP synthase. We tested the hypothesis that markerless polar mutations could be efficiently engineered using CRISPR/Cas12a in these loci.

**Results:**

Cas12a-mediated engineering of a terminator sequence inside the *fimA* gene occurred with efficiencies between 10 and 80% and depended on the terminator’s sequence, whilst other types of mutations, such as a 97 bp deletion, occurred with 100% efficiency. Polar mutations using a terminator sequence were also engineered in the *atp* locus, which induced its transcriptional shutdown and produced identical phenotypes as a deletion of the whole *atp* locus (Δ*atpIBEFHAGDC*). Measuring the expression levels in the *fim* and *atp* loci showed that many supposedly non-polar mutants induced a significant polar effect on downstream genes. Finally, we also showed that transcriptional shutdown or deletion of the *atp* locus induces elevated levels of intracellular ATP during the exponential growth phase.

**Conclusions:**

We conclude that Cas12a-mediated mutagenesis is an efficient simple system to generate polar mutants in *E. coli*. Different mutations were induced with varying degrees of efficiency, and we confirmed that all these mutations abolished the functions encoded in the *fim* and *atp loci.* We also conclude that it is difficult to predict which mutagenesis strategy will induce a polar effect in genes downstream of the mutation site. Furthermore the strategies described here can be used to manipulate the metabolism of *E. coli* as showcased by the increase in intracellular ATP in the markerless Δ*atpIBEFHAGDC* mutant.

**Supplementary Information:**

The online version contains supplementary material available at 10.1186/s12934-022-01844-y.

## Background

The evolutionary success of polycistronic operons has been attributed to their contribution to the organization of metabolic pathways, which ultimately allowed organisms to become less dependent on exogenous sources of organic compounds [1]. Importantly, this relationship was maintained during evolution for many metabolic pathways and was probably enhanced by horizontal gene transfer [1]. In *E. coli*, 63% of its genes are organized into polycistronic operons [2], and 40% are organised into so-called uber operons [3]. At both organisation levels, genes tend to be related by functional conservation across bacterial genomes [3–5]. Genes within polycistronic transcriptional units are co-transcribed from a single promoter, but it has been shown that the mRNA of each open reading frame (ORF) has an independent folding from their neighbouring genes, and this feature may directly influence its translation efficiency [6]. Other features regulating operon expression and function in *cis* include attenuators, terminators and processive antiterminators (reviewed in [7, 8]). All these elements of regulation depend on the formation of secondary RNA structures. We aimed to test whether sequences with such a tendency to generate secondary structures could be readily engineered in the *E. coli* chromosome using CRISPR/Cas technologies. CRISPR/Cas genome editing in *E. coli* [9] opened the possibility of marker-less genetic engineering with single base resolution. Therefore, it is possible to generate multi-scale libraries of mutants in a few days [10]. The objective of the following study was to design a strategy to shut down the transcription of entire polycistronic operons using synthetic terminator sequences [11] in *E. coli*.

Type 1 fimbriae are adhesive surface appendages present mainly in *Enterobacteriaceae* that were first shown to agglutinate red-blood cells [12]. They are composed of a major fimbrial subunit (FimA) repeated on the order of hundreds to thousands and are assembled at the surface by an outer membrane usher. At the tip of the filament, the so-called minor pilins are assembled in a specific order. The most distal pilin to the cell (FimH) functions as an adhesin [13, 14]. In the case of *E. coli* type 1 fimbriae, the FimH adhesin interaction with red blood cells is inhibited by soluble D-mannose residues or α-methyl-mannoside that compete out the recognition of mannosylated receptors present on mammalian cells [15]. Its role in virulence in uropathogenic strains of *E. coli* has been established for a long time [16, 17]. However, its role in persistent colonization of the mammalian intestine has only recently been shown to be essential for the case of uropathogenic *E. coli* [18]. It has not yet been clarified how each of the fimbrial systems present in symbiotic *E. coli* strains [19] contributes to intestinal colonization in vivo. In this study, we tested the effects of introducing markerless mutations in the *fimA* locus of the *fimAICDFGH* operon, and we studied their polar effect on the transcription of downstream genes.

As an additional test of the polar mutagenesis strategy, we genetically modified the *atp* locus, which codes for the FoF1 ATP synthase [20]. Adenosine 5′-triphosphate (ATP) is the nucleotide derivative that stores chemical energy, and many enzymes use this energy made available by breaking ATP into adenosine 5′-diphosphate (ADP). The FoF1 ATP synthase complex catalyses the interconversion of ATP and ADP and is the primary source of ATP in many organisms together with fermentative glycolysis. The intracellular level of ATP is a critical level for bio-production (reviewed in [21]). Therefore, we hypothesised that disrupting the *atp* locus would create a measurable way to alter cellular metabolism.

We showed in this study that genome editing with CRISPR/Cas12a engineering can be readily used to shutdown the transcription of whole polycistronic transcriptional units, and we studied how some mutagenesis strategies can induce polar effects. Modifying or deleting the *atp* operon, we also showed that this approach can lead to the manipulation of *Escherichia coli*’s metabolism to increase intracellular levels of ATP.

## Results

### Mutagenesis of the *fimA* gene

**Fig. 1:**
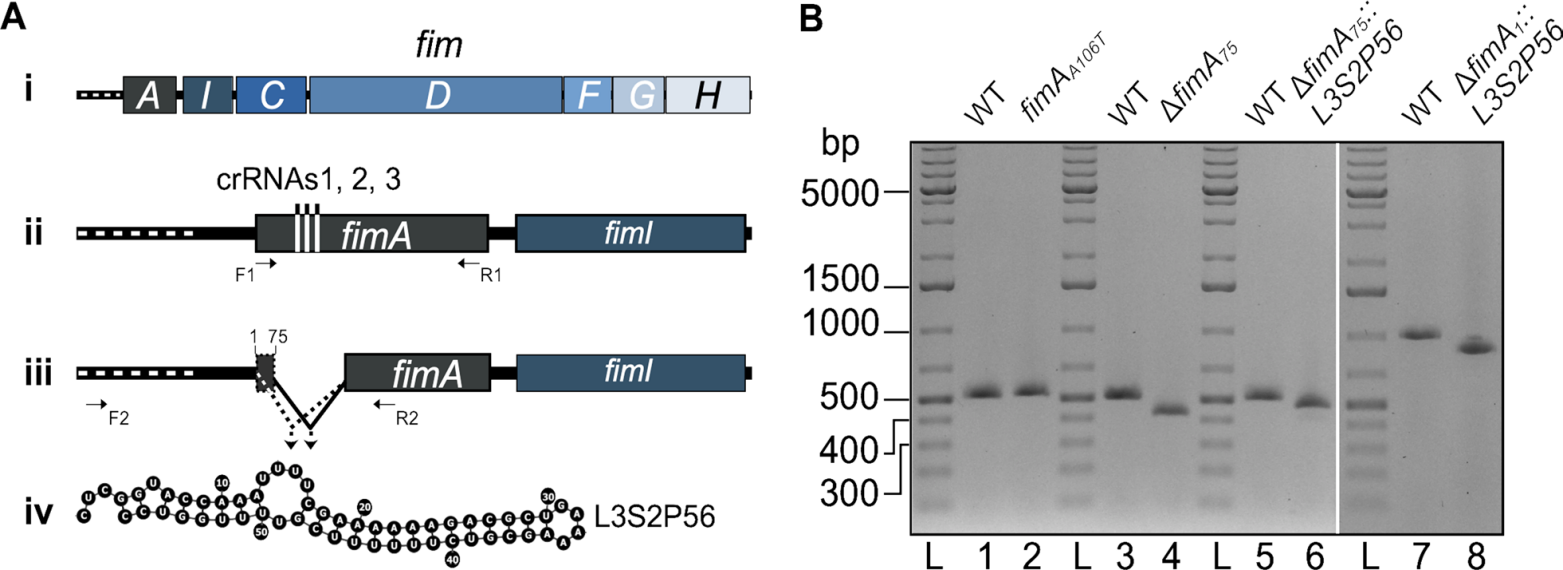
Design of Cas12a-dependent mutagenesis of the *fimAICDFGH* operon. **A** Scheme of the *fimAICDFGH* operon (**i**). Its transcription depends on a promoter (*fimAp*) that is part of an invertible DNA element indicated by a white dotted line upstream of *fimA* (**ii**). The target sites for Cas12a crRNA binding sites in *fimA* are shown as horizontal lines (**ii**). The site of the 97 bp deletion at position 75 of the *fimA* gene is illustrated as a gap (**iii**). Positions 1 and 75 in *fimA* where the L3S2P56 terminator sequence was inserted to generate mutants Δ*fimA*_*1*_*::L3S2P56* and Δ*fimA*_*75*_*::L3S2P56* are marked on **iii**. The lines converging on an arrow denote the regions spanning the L3S2P56 terminator sequence insertions (**iii**). The ribonucleotide sequence and expected folding of the RNA corresponding to the transcriptional terminator L3S2P56 (**iv**)*.* **B** Analysis of *fimA* mutants by agarose gel electrophoresis of PCR products. Lanes 1, 3, and 5, show a 514 bp PCR product generated by primers F1 and R1 (shown in **ii**) that amplify a fragment of *fimA* from wild type *E. coli*. Lane 2 shows the unchanged migration pattern of the PCR product compared to WT using primers F1 and R1 on template DNA from the stop codon mutant (*fimA*_*A106T*_). Lanes 4 and 6 show the PCR product generated using primers F1 and R1 after amplifying template DNA from the deletion mutant (Δ*fimA*_*75*_, 420 bp) and the internal terminator insertion (Δ*fimA*_*75*_*::L3S2P56*, 474 bp), respectively. Lane 7 shows a 988 bp PCR product generated by primers F2 and R2 (shown in **iii**) that amplify a fragment of *fimA* from wild type *E. coli* including the leader sequence and lane 8 shows the 870 bp PCR product generated with primers F2 and R2 on template DNA from the second terminator insertion (Δ*fimA*_*1*_*::L3S2P56*). Lane L was loaded with a double-stranded DNA ladder

We set out to disrupt the *fimAICDFGH* operon in *E. coli* K-12 using Cas12a to introduce a range of different markerless mutations in the *fimA* gene, including the insertion of terminator sequences (Fig. 1A). We designed a CRISPR RNA (crRNA) targeting *fimA*. To introduce polar and non-polar mutations in the *fimAICDFGH* operon, we designed two complementary oligonucleotides for each mutation to form dsDNA donor molecules for homologous recombination and Cas12a-mediated positive selection of mutant cells [22]. Four mutations were designed using this strategy. The first mutation introduced a premature stop codon in the *fimA* gene by substituting a single base (*fimA*_*A106T*_) and the second mutation was a deletion of 97 bp at residue 75 inside the gene (Δ*fimA*_*75*_). To engineer a polar mutagenesis strategy, we designed two different donor oligonucleotides with homology arms that inserted a 57 bp terminator sequence in the *fimA* gene at residues 1 (*fimA*_*1*_) and 75 (*fimA*_*75*_) beside a 97 and a 172 bp deletion, respectively (Fig. 1).

Terminator sequences have a strong tendency to form secondary structures, as illustrated by a set of terminator transcripts shown in Additional file 1: Fig. S1. We chose to use the terminator sequence L3S2P56 [11] and set out to determine if such a sequence could serve as a DNA donor for CRISPR/Cas12a mutagenesis. To test this hypothesis, we simply electroporated *E. coli* cells carrying the plasmid pKD46-Cas12a [22] with the annealed dsDNA donor oligonucleotides and a plasmid encoding crRNA1. We analysed transformants by colony PCR using primers that amplify a segment of the *fimA* gene surrounding the site of crRNA targeting. For the stop codon mutant (*fimA*_*A106T*_) obtained with crRNA1, we analysed five colonies. The size of the PCR fragment did not appear to be changed compared to wild type (WT) *E. coli* (Fig. 1B). We purified these PCR products and submitted them for Sanger sequencing. Four of the five candidates showed the specific T- > A substitution that introduces a premature stop codon. For the Δ*fimA*_*75*_ mutant allele, the PCR fragment showed a noticeable change in migration pattern compared to the WT *fimA* allele (Fig. 1B). Ten out of ten colonies picked showed the deletion (Additional file 1: Fig. S2). For mutants carrying the alleles with the insertion of a terminator sequence Δ*fimA*_*75*_*::L3S2P56* and Δ*fimA*_*1*_*::L3S2P56*, only three out of ten and one out of ten colony PCRs tests showed a change in migration pattern, respectively (Fig. 1 and Additional file 1: Fig. S2). Altogether, these results indicate that the engineering of terminator sequences is possible but with lower success rates compared to the highly efficient engineering of single base substitutions or deletions.

### Phenotypic analysis of *fimA* mutants

**Fig. 2:**
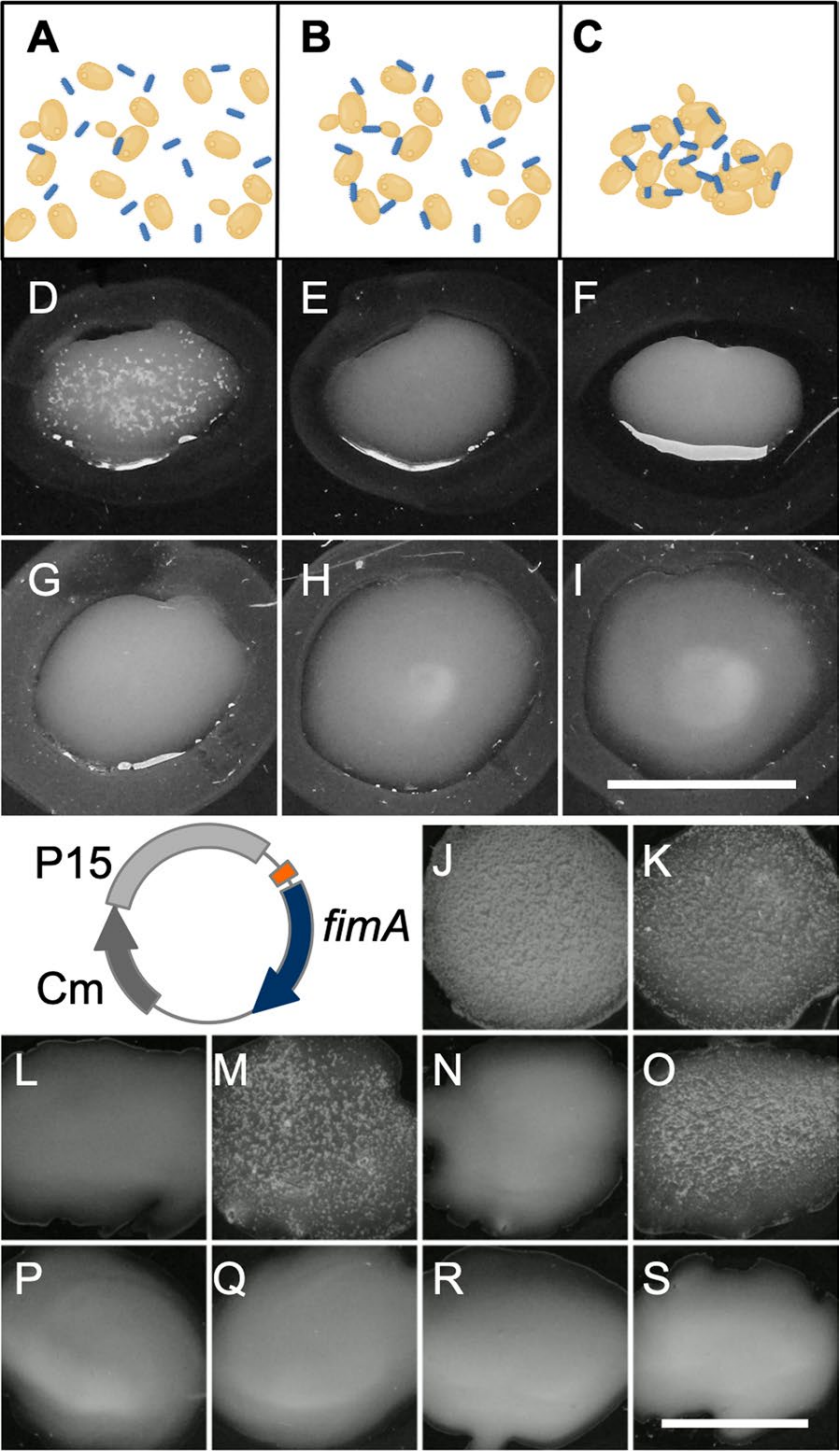
Agglutination assay of *fimA* mutant bacterial derivatives. A schematic representation of the agglutination assay: **A** A suspension of *Saccharomyces cerevisiae* cells (yellow) was spread together with bacteria (blue) on a glass plate. **B** Bacteria adhering to yeast cells by virtue of the FimH fimbriae subunit that recognizes mannose residues on the surface of yeast cells. **C** The polyvalent binding of fimbriated bacteria to yeast cells causes an agglutination chain reaction, which is macroscopically visible. Photographic images of agglutination reaction tests performed with the following bacterial strains: **D** WT *E. coli*, **E**) *Shigella flexneri* M90T (negative control), **F** Stop codon mutant (*fimA*_*A106T*_) *E. coli*, **G** *E. coli* Δ*fimA*_*75*_, **H** *E. coli* Δ*fimA*_*75*_*::L3S2P56 and* **I** *E. coli* Δ*fimA*_*1*_*::L3S2P56.* Plasmid trans-complementation of *fimA* mutant derivatives. As illustrated in the schematic circular plasmid map, the *fimA*^+^ *allele* was expressed from a P15 plasmid derivative under control of the *lac* promoter (orange). The empty vector, pSU19, was used as negative control. Agglutination reaction for the following strains: **J** WT *E. coli* + empty vector, **K** WT *E. coli* + *fimA*^+^ plasmid, **L** *E. coli fimA*_*A106T*_ + empty vector, **M** *E. coli fimA*_*A106T*_ + *fimA*^+^ plasmid, **N** *E. coli* Δ*fimA*_*75*_ + empty vector, **O** *E. coli* Δ*fimA*_*75*_ + *fimA*^+^ plasmid, ***P E. coli*** Δ*fimA*_*75*_*::L3S2P56* + empty vector, **Q** *E. coli* Δ*fimA*_*75*_*::L3S2P56* + *fimA*^+^ plasmid, **R** *E. coli* Δ*fimA*_*1*_*::L3S2P56* + empty vector, **S** *E. coli* Δ*fimA*_*1*_*::L3S2P56* + *fimA*^+^ plasmid*.* Scale bars **D**–**S**: 2 cm. **A**–**C** created with BioRender.com

To characterise the effect of the different mutations, we verified the functionality of the *fimAICDFGH* gene products by a phenotypic assay. We performed agglutination assays using *Saccharomyces cerevisiae* cells (Fig. 2A–C). Overnight cultures of WT and *fimA* mutant *E. coli* strains were mixed with a drop of a yeast cell suspension on a glass plate. With WT *E. coli*, this led to agglutination of the yeast cells, which appeared as white, macroscopically visible aggregates (Fig. 2D). Contrastingly, mixing *Shigella flexneri* M90T, which is known not to produce mannose-binding fimbriae when grown in tryptic soy agar [23], caused no yeast aggregates (Fig. 2E). Mixing *E. coli* carrying each of our *fimA* mutant alleles with the yeast cell suspension produced no agglutination (Fig. 2F–I). There was no difference between the stop codon (*fimA*_*A106T*_), deletion (Δ*fimA*_*75*_) and terminator mutant alleles (Δ*fimA*_*75*_*::L3S2P56* and Δ*fimA*_*1*_*::L3S2P56*), which suggests that the disruption of even one base introducing a stop codon was enough to convert these strains to a Fim negative phenotype.

To verify the efficiency of the different mutations in exerting a polar effect, we tested the functionality of the *fimAICDFGH* operon by a phenotypic assay in WT and mutant strains carrying a plasmid expressing the *fimA*^+^ allele (Fig. 2). Each strain was transformed with the trans-complementing plasmid and an empty plasmid vector as a negative control. *E. coli* carrying the wild type *fimA* allele and an empty vector agglutinated *S. cerevisiae* as expected. Similarly, the expression of plasmid-encoded FimA^+^ had no effect on the agglutination reaction (Fig. 2J, K), suggesting that neither of the plasmids had an impact on agglutination. Trans-complementation of bacteria carrying both the stop codon and *fimA* deletion mutant alleles resulted in yeast cell agglutination (Fig. 2M and O), suggesting that these mutations caused little or no effect on the downstream genes that code for the assembly machinery and surface localisation of the FimH adhesin. As expected, the strains carrying the empty vector were agglutination negative (Fig. 2L and N). Trans-complementation tests of bacteria carrying both the Δ*fimA*_*75*_*::L3S2P56* and Δ*fimA*_*1*_*::L3S2P56* mutant alleles with neither the empty plasmid (Fig. 2P and R) nor the plasmid-encoded FimA^+^ (Fig. 2Q and S) resulted in yeast cell agglutination. This suggests that the functionality of the *fimAICDFGH* operon was abolished due to disrupted expression of genes downstream of *fimA* in these two mutants. We conclude that the introduction of the L3S2P56 terminator functionally disrupted the transcription of the entire *fim* operon.

### Single cell analysis of a *fimA* terminator mutant by AFM

**Fig. 3:**
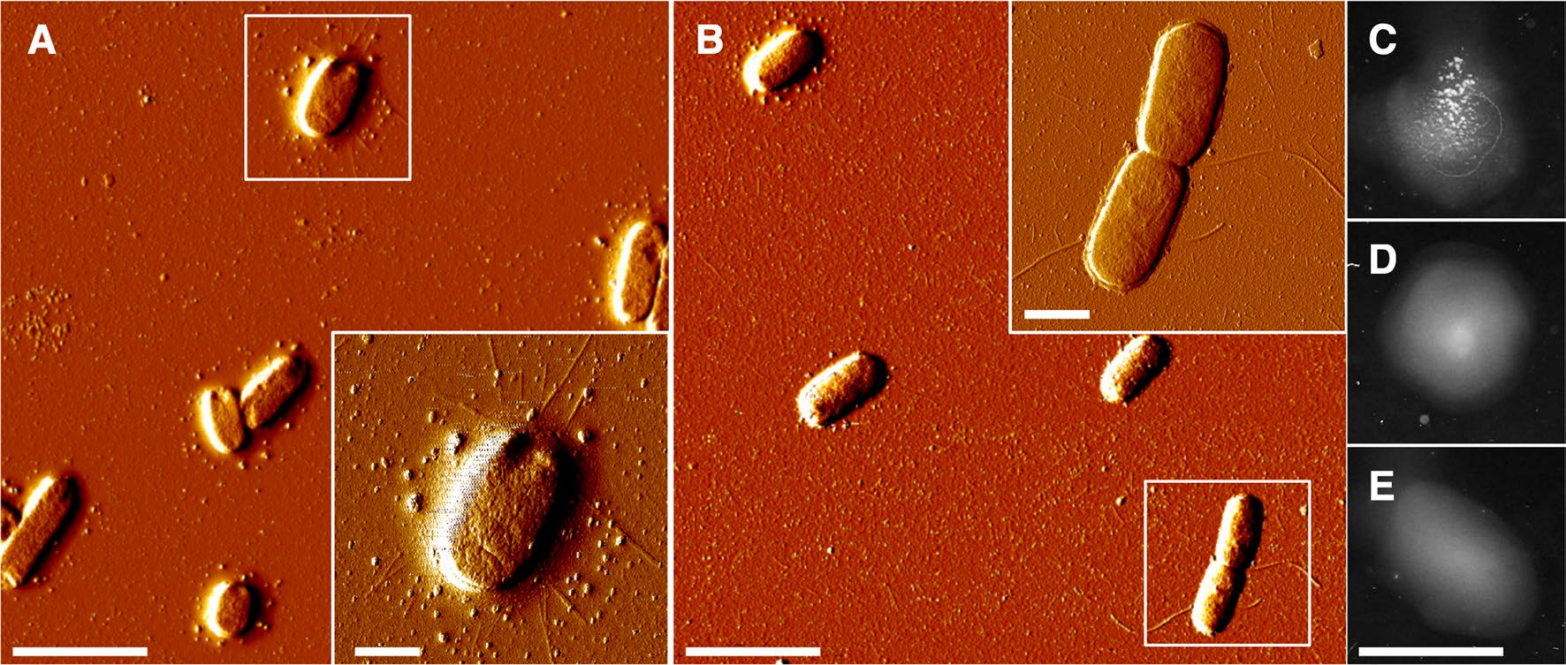
Atomic force microscopy (AFM) and agglutination tests with bacteria from static cultures. **A** AFM imaging of wild-type *E. coli* + *fimA* plasmid grown in a static liquid culture. **A** *E. coli* cell with type 1 fimbriae is shown in the inset. **B** AFM imaging of the Δ*fimA*_*1*_*::L3S2P56* mutant *E. coli* + *fimA*^+^ plasmid grown in a static liquid culture. An *E. coli* cell with flagella is shown as an inset. **C** Yeast agglutination assay of wild-type *E. coli* + *fimA*^+^ plasmid grown in a static liquid culture. **D** Yeast agglutination assay in the presence of α-methyl-mannoside of of wild-type *E. coli* + *fimA*^+^ plasmid grown in a static liquid culture in the presence of α-methyl-mannoside. **E** Yeast agglutination assay of the Δ*fimA*_*1*_*::L3S2P56* mutant *E. coli* + *fimA*^+^ plasmid grown in a static liquid culture. Scale bars: **A, B** 4 μm. **A**, **B** Insets 1 μm. **C**–**E** 2 cm

We wanted to directly observe fimbriae formation, so we monitored the bacterial cells by Atomic Force Microscopy (AFM). Earlier fluorescence microscopy measurements have shown an increase in fimbriae formation in liquid static cultures of *E. coli* strain MG1655 [24]. Therefore, we grew *E. coli* with the wild type *fimA* and Δ*fimA*_*1*_*::L3S2P56* mutant alleles that also expressed the *fimA*^+^ allele from a plasmid as static cultures to observe whether there was any formation of fimbriae. Overnight cultures were prepared on freshly cleaved mica for AFM observation. In the wild type strain carrying the plasmid-encoded *fimA*^+^ allele, we observed bacteria displaying the classical type 1 fimbriae (Fig. 3A and A inset). However, in the case of bacteria with the Δ*fimA*_*1*_*::L3S2P56* mutant allele, we did not observe any formation of fimbriae despite that it was carrying a plasmid-encoded *fimA*^+^ allele (Fig. 3B). On some bacterial cells, only the formation of flagella could be observed (Fig. 3B inset). As a control, we performed AFM imaging of the Δ*fimA*_*75*_ mutant carrying the transcomplementation empty and FimA^+^ plasmids grown as static cultures. Only *E. coli* Δ*fimA*_*75*_ carrying the *fimA*^+^ allele showed type 1 fimbriae (Additional file 1: Fig. S3). The results from tests with the yeast cell agglutination assay also confirmed the presence or absence of functional fimbriae (Fig. 3E). The wild type strain expressing plasmid-encoded *fimA*^+^ allele was agglutination-positive, whereas the addition of α-methyl-mannoside completely abolished agglutination, confirming the mannose-specificity of the agglutination reaction (Fig. 3C, D). The *fimA*^+^ trans-complemented Δ*fimA*_*1*_*::L3S2P56* mutant strain was also agglutination-negative after growth under static growth conditions (Fig. 3E). All these results confirm that the Δ*fimA*_*1*_*::L3S2P56* mutant allele gives a Fim negative phenotype even when the *fimA*^+^ allele is expressed in *trans*.

### Polar effects of *fimA* mutations

**Fig. 4:**
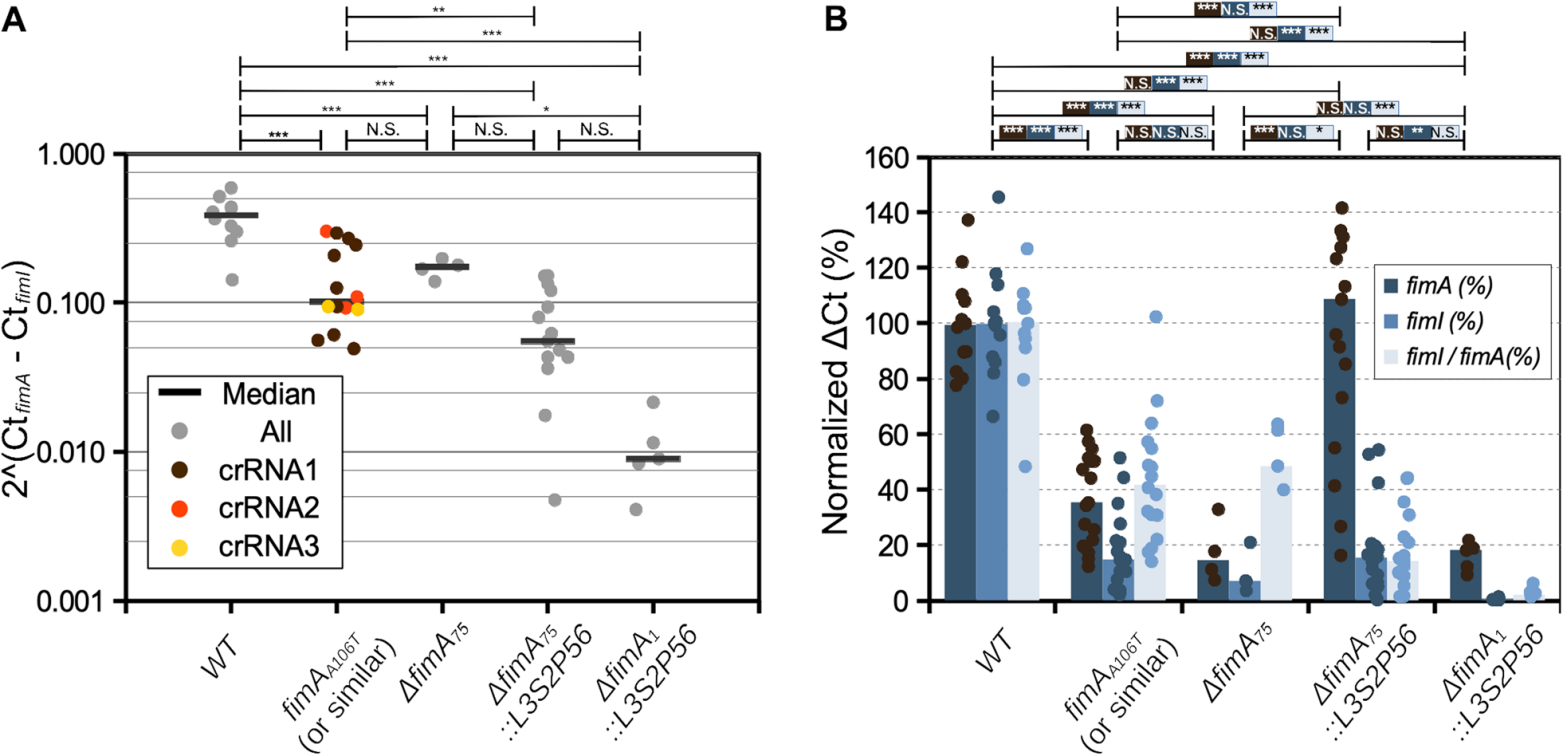
Quantitative PCR analysis of *fimA* and *fimI* expression in different* E. coli* derivatives. **A** *fimI* expression normalized to *fimA* expression. Each data point represents a biological replicate and the median is shown as a horizontal bar. **B** Same data used in **A** but represented as median ΔCt expression of *fimA* and *fimI* normalized to the housekeeping genes *hcaT* and *cysG* and expressed as percent of the wild type. The statistical analysis shows the results of a one-way ANOVA (panel **A** and %ΔCt *fimI*/*fimA* ratio in panel **B** or two-way ANOVA (%ΔCt of *fimI* and *fimA* in panel **B** followed by Tukey's HSD multiple comparison tests. P values: *P* ≤ 0.001 (***), *P* ≤ 0.01 (**), *P* ≤ 0.05 (*), *P* ≤ 0.1 (0.1), *NS* not significant

To quantify the extent of a polar effect in each of the *fimA* mutants, we used qPCR to measure the levels of *fimA* and *fimI* mRNA. We designed two sets of primers that allowed for the estimation of the levels of transcripts from these two genes. We performed qPCR using as template cDNA derived from mRNA obtained from *E. coli* cultures grown in the same conditions as those used for the phenotypic assays (Fig. 2). Initially, we estimated the amount of *fimI* mRNA relative to *fimA.* This would represent an unbiased measurement that does not depend on the ON/OFF state of the *fimAICDFGH* operon expression that is subject to phase variation due to the invertible DNA element that includes the transcriptional promoter *fimAp* [25]*.* The relative expression of *fimI* in wild type *E. coli* was  ~ 2.5 times lower than that of *fimA*. The median relative expression of *fimI* in the stop and deletion mutants was significantly lower but on the same order of magnitude (Fig. 4A). The median relative expression of *fimI* in the Δ*fimA*_*75*_*::L3S2P56* and Δ*fimA*_*1*_*::L3S2P56* mutants was significantly lower and on different orders of magnitude compared to the wild type (Fig. 4A). The relative expression fold change for the Δ*fimA*_*75*_*::L3S2P56* and Δ*fimA*_*1*_*::L3S2P56* mutants was  ~ 9- and  ~ 43-fold, respectively. These results suggest that at least an estimated nine-fold change in expression is sufficient to disrupt the functionality of the *fim* operon downstream of *fimA*.

We also measured the levels of *fimI* and *fimA* mRNA levels with respect to housekeeping genes. We designed sets of primers for the housekeeping genes *hcaT* and *cysG*, which have been shown to have stable expression [26]. We obtained the average ΔCt with respect to these two genes and normalized it to the average value of the WT control. The levels of *fimA* were significantly lower in three out of four mutants, and the levels of *fimI* significantly decreased in all four mutants. *E.* coli with the stop codon, the deletion and the Δ*fimA*_*1*_*::L3S2P56* mutant alleles had lower levels of *fimA* mRNA than the WT (Fig. 4B). Inexplicably, bacteria with the Δ*fimA*_*75*_*::L3S2P56* mutant allele differed from other *fimA* mutant strains and had median levels of *fimA* mRNA comparable to wild type, although with higher variability. (Fig. 4B). The levels of *fimI* were significantly lower for all the mutants, with the Δ*fimA*_*75*_*::L3S2P56* and Δ*fimA*_*1*_*::L3S2P56* mutants having the most severe reduction (Fig. 4B).

As described above, we found a significant difference between the wild type and the stop codon mutant (*fimA*_*A106T*_) in the *fimI* mRNA levels relative to *fimA* (Fig. 4A). To further investigate this phenomenon, we created two more stop codon mutants. We co-transformed *E. coli* with plasmids encoding crRNA2 or crRNA3 (Fig. 1) with donor template oligonucleotides introducing a stop codon instead of the PAM sequences into *E. coli* carrying the plasmid pKD46-Cas12a. For crRNA2, the donor oligonucleotides introduced the mutations T103A and T104A (*fimA*_*T103A-T104A*_) to disrupt the PAM sequence. For crRNA3, the donor oligonucleotide introduced the mutations A158T, A159T, A160T, C161A, and C162A (*fimA*_*A158T-A159T-A160T-C161A-C162A*_). Two clones of each of these mutants were selected and analysed by qPCR measurements of *fimI* and *fimA* mRNA levels*.* The median relative expression of *fimI* to *fimA in* these two new mutants was also lower but on the same order of magnitude as for mutants carrying the allele *fimA*_*A106T*_ (Fig. 4A, orange and yellow circles). This result suggests that the reduced levels in the amount of *fimI* mRNA relative to *fimA* expression are consistent in all three stop codon mutants and are not caused by an off-target effect of crRNA1.

### The mutagenesis efficiency depends on the sequence of the donor oligonucleotides

**Fig. 5:**
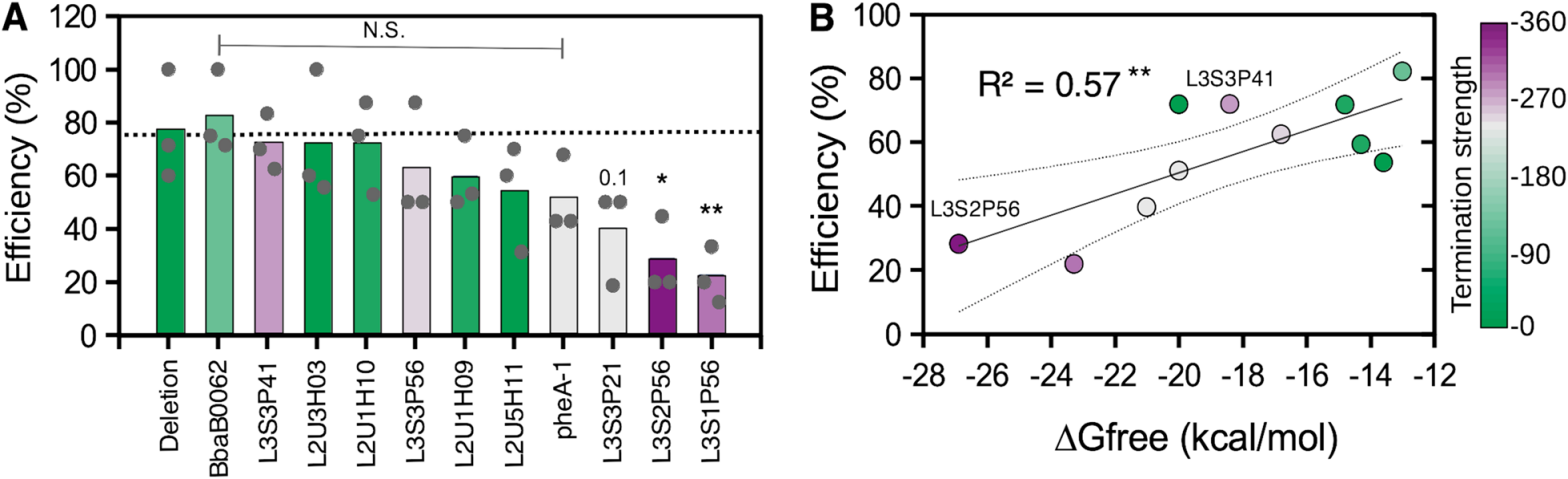
Effect of the terminator sequences on mutagenesis efficiency. **A** Average mutagenesis efficiency expressed as the percentage of colonies identified as mutants by colony PCR genotyping for every terminator sequence tested in three independent biological replicates. The colour code (scale bar shown on the right side of panel **B** represents the termination strength as determined by Chen et al. [11]. The statistical analysis shows the results of a one-way ANOVA followed by a Holm-Sidak’s multiple comparisons test comparing all values to the control sample (deletion oligonucleotide). P values: *P* ≤ 0.001 (***), *P* ≤ 0.01 (**), *P* ≤ 0.05 (*), *P* ≤ 0.1 (0.1), N.S. (not significant). **B** Average mutagenesis efficiency represented as colour-coded percentage of colonies identified as mutants by colony PCR genotyping in correlation to the ΔGfree of the secondary structure formation of the terminator sequences as calculated by the Vienna RNA suite [27]. The curved dotted solid lines represent the 95% confidence interval of the linear regression (solid line). P value of R^2^: *P* ≤ 0.01 (**)

We wanted to compare whether other terminator sequences also had decreased mutagenesis efficiency. We designed complementary oligonucleotides for each mutation to form dsDNA donor molecules for homologous recombination and Cas12a-mediated positive selection of mutant cells [22]. The dsDNA molecules had different terminator sequences but had the exact same homology recombination donor arms as the Δ*fimA*_*75*_ and Δ*fimA*_*75*_*::L3S2P56* mutants. Therefore, we were able to observe the effect of the terminator sequences on the mutagenesis efficiency. To obtain insight into different terminator properties, we chose ten new terminator sequences from the Chen et al. dataset [11] with different terminator strengths as the sole criterion (Fig. 5 and Additional file 1: Fig. S1). *E. coli* cells carrying the plasmid pKD46-Cas12a [22] were transformed with the different annealed dsDNA donor oligonucleotides and a plasmid encoding crRNA1. The next day, colonies were picked and genotyped as above to determine the ratio of WT to mutant cells and calculate the mutagenesis efficiency. Our results showed that the average mutagenesis efficiencies of the different donor dsDNA molecules containing the different terminator sequences ranged from  ~ 20 to  ~ 80% (Fig. 5). One of the terminator sequences with significantly low efficiency in this new experiment was the L3S2P56 terminator sequence, which had already been characterised as having low efficiency (Fig. 1 and Additional file 1: Fig. S2). Interestingly, we primarily chose this terminator due to its high termination strength. Another terminator sequence with significantly low mutagenesis efficiency, namely, L3S1P56, had also been characterized as having high termination strength [11]. Moreover, the highest efficiencies were observed for terminator sequences with low or intermediate termination strength (Fig. 5A, colour code and Additional file 1: Fig. S1) [11]. Therefore, our results suggested a potential link between the termination strength and the mutagenesis efficiency. One exception was the L3S3P41 terminator sequence (Additional file 1: Fig. S1), which showed a high efficiency but has been characterized as providing strong termination. To visualise the link between the termination strength and the mutagenesis efficiency, we colour coded the termination strength as determined by Chen et al. [11]. Indeed, the 3 terminators with the significantly lowest efficiencies (L3S2P56, L3S1P56, LRS3P21) were previously characterized to have the highest, 2nd-highest and 4th-highest termination strengths [11] from our subset (Fig. 5, colour code and Additional file 1: Fig. S1). To determine if the thermodynamic stability of the terminator sequences is linked to the mutagenesis efficiency, we plotted it against the free energy change from the secondary structure (ΔGfree) as calculated by the Vienna RNA suite [27]. The correlation coefficient was weak but positive (R^2^ = 0.57), suggesting that the secondary structure of the terminator sequences might influence the recombination capacity of the donor dsDNA oligonucleotides. When we analysed the correlation of other thermodynamic properties from each terminator sequence as determined by Chen et al. (Additional file 1: Fig. S4) with the mutagenesis efficiency, the property that has the highest correlation was ΔGA, which corresponds to the free energy of the extended hairpin (R^2^ = 0.23). However, the length of the terminator had a higher correlation (R^2^ = 0.35). Interestingly, the exception to the rule, L3S3P41, which is a terminator that has been characterized to have high termination strengths, is one of the shortest in our subset but has a medium–high ΔGfree (Additional file 1: Fig. S1). All of these results show that to introduce potential artificial termination, there are sequences that can lead to significantly higher mutagenesis efficiency than others independent of the recombination homology arms and the crRNA.

### Polar mutagenesis of the *atpBEFHAGDC* locus

**Fig. 6:**
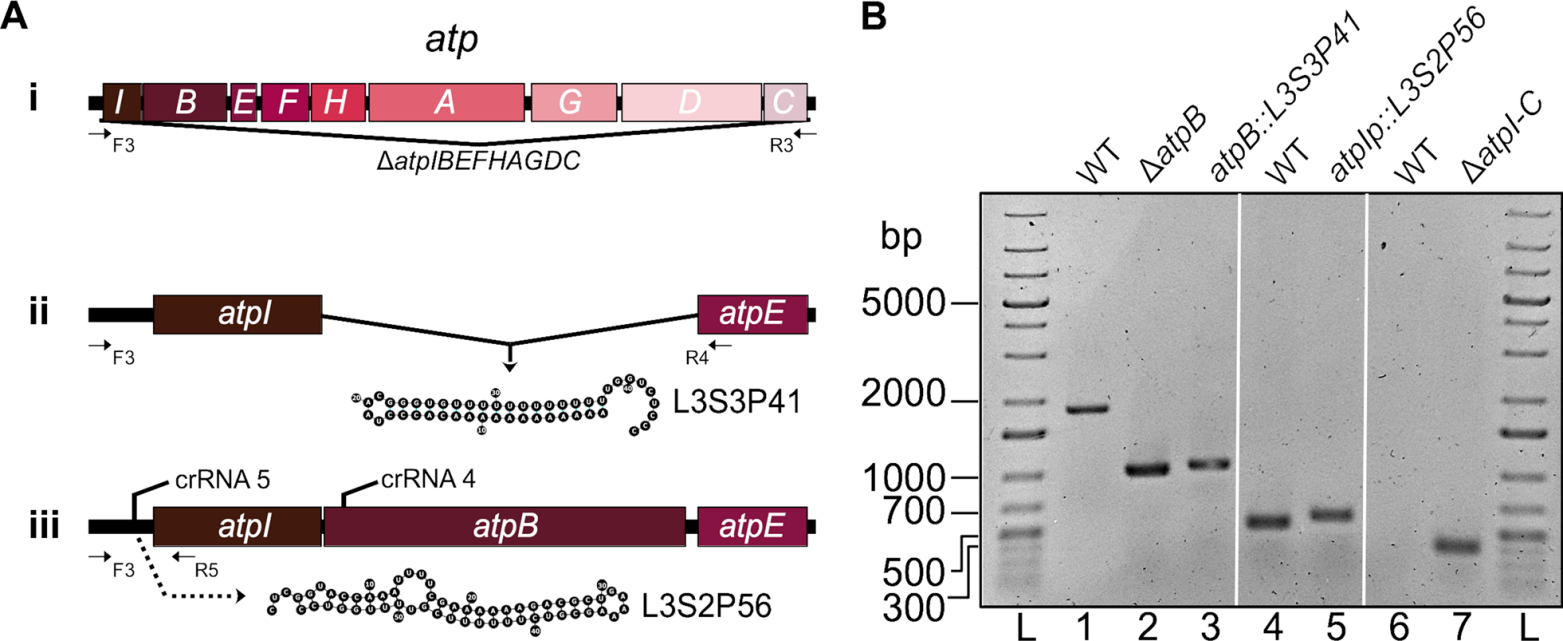
Design of Cas12a-dependent mutagenesis of the *atpIBEFHAGDC* operon. **A** Scheme of the *atpIBEFHAGDC* operon (**i**). The 816 bp deletion of the *atpB* gene is illustrated as a gap and the lines converging on a vertical arrow denote the L3S3P41 insertion at that position to generate mutant *atpB::L3S3P41* (**ii**). Included here is also the ribonucleotide sequence and expected folding of the RNA corresponding to the L3S3P41 transcriptional terminator (**ii**). The insertion of the L3S2P56 terminator sequence upstream of *atpI* and downstream of the *atpIp* promoter that generated the *atpIp::L3S2P56* mutant is illustrated as a dotted arrow (**iii**)*.* The target sites for Cas12a crRNA binding sites in *atpIp* and *atpB* are shown as horizontal lines (**iii**). Horizontal arrows indicate positions corresponding to DNA primers used in PCR analyses, here named F3, R3, R4 and R5. (**i**, **ii**, **iii**). **B** Analysis of *atp* mutants by agarose gel electrophoresis of PCR products. Lane 1 shows the 1837 bp PCR product generated by primers F3 and R4 that amplify a segment of the genes *atpIB* in genomic DNA from wild type *E. coli.* Lane 2 shows the migration pattern of the 1021 bp and 1068 bp PCR products on DNA from the *E. coli atpB* and *atpB::L3S3P41* mutants generated by primers F3 and R4, respectively. Lanes 4 and 5 show a 521 and 575 bp PCR products generated by primers F3 and R5 that amplify a segment of the promoter *atpIp* and the *atpI* gene on DNA from the WT *E. coli* and *atpIp::L3S2P56* mutants, respectively. Lane 7 shows a PCR fragment produced by primers F3 and R3 that amplify a 385 bp product present only in the *E. coli* Δ*atpIBEFHAGDC* mutant but not in the WT strain (lane 6). Lane L was loaded with a double-stranded DNA ladder

We wanted to study the effect of introducing polar and non-polar mutations in a different polycistronic transcriptional unit, and for this we studied the *atpBEFHAGDC* locus, which codes for the FoF1 ATP synthase complex. It has previously been shown that such a mutant could be constructed in *E. coli* [28]*.* We designed crRNAs matching sequences corresponding to the main promoter of the transcriptional unit (*atpIp*) and the *atpB* gene (Fig. 6A). As in the case of the *fim* locus, we designed complementary oligonucleotides for each mutation to form dsDNA donor molecules for homologous recombination and Cas12a-mediated positive selection of mutant cells [22]. Four mutants were engineered: a deletion of the *atpB* gene (Δ*atpB*), a substitution of the *atpB* gene for the L3S3P41 terminator sequence (*atpB::L3S3P41*), an insertion of the L3S2P56 terminator sequence between the *atpIp* promoter and the start codon of the *atpI* gene (*atpIp::L3S2P56*) and finally a deletion of the whole locus (Δ*atpIBEFHAGDC*). The latter mutant comprises a deletion of 7034 bp, which was easily achieved using a 110 bp dsDNA donor oligonucleotide. The first three mutants were genotyped by PCR and agarose electrophoresis using specific primers that annealed upstream of *atpIp* and in the middle of either the *atpI* gene or *atpE gene* (Fig. 6A, B). The complete locus deletion was genotyped using primers that annealed upstream of the *atpIp* promoter and downstream of the *atpC* gene, which showed an amplified fragment of 385 bp present only in the mutant strain (Fig. 6B).

**Fig. 7:**
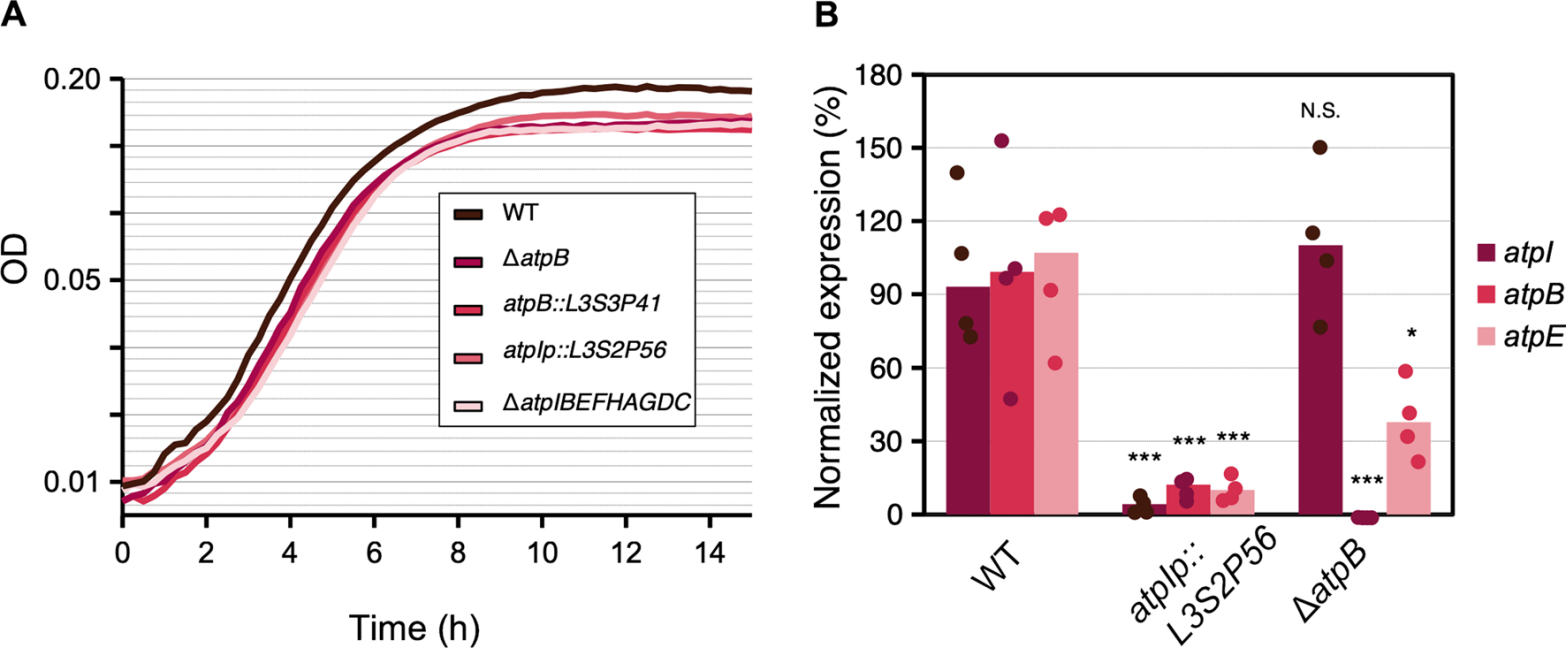
Phenotypic characterization of *atp mutants*. **A** Growth curves of *atp* mutants in minimal medium supplemented with glucose. **B** Normalized expression analysis by qPCR of *atpI*, *atpB* and *atpE* after three hours of growth*.* The ΔCt expression values were normalized to *hctA* and *CysG* and represented as the median percentage of expression of each gene in the WT strain. The statistical analysis shows the results of a two-way ANOVA (panel **B**) followed by Tukey’s HSD multiple comparison tests. P values: *P* ≤ 0.001 (***), *P* ≤ 0.01 (**), *P* ≤ 0.05 (*), *NS* not significant

We first explored the effect of these mutations on their ability to grow in minimal medium supplemented with glucose, as previously shown [28]. We found a difference in growth between the wild type strain and all the mutants manifested as a difference in the obtained yield, i.e., the maximum density that the cultures reached in stationary phase (Fig. 7A). However, there was no apparent difference in growth among any of the *atp* mutants. Moreover, there was no apparent change in the growth rate during the exponential phase. Of note, the *atpIp::L3S2P56* insertion mutant, which has no deletion of any *atp* gene, had the same phenotype as the *atpB* deletion and insertion mutants and the Δ*atpIBEFHAGDC* mutant.

We also measured the expression of the genes *atpI*, *atpB*, and *atpE*. We performed qPCR with specific probes of cDNA produced from cultures grown for three hours in minimal medium supplemented with glucose. For this experiment, we compared the wild type strain with the mutant that has an L3S2P56 insertion downstream of the *atpIp* promoter and a deletion of the *atpB* gene. Our results showed that the Δ*atpB* mutant had expression levels of *atpI* at the same level as that of the wild type. However, the levels of expression of the *atpE* gene were  ~ 40% compared to WT, indicating a small but statistically significant polar effect of the *atpB* deletion on the expression of *atpE*, although not completely abolishing it. As expected, we could not detect any trace of the *atpB* gene (Fig. 7B). When we analyzed the expression levels of *atpIBE* in the *atpIp::L3S2P56* insertion mutant, we observed that the expression levels of all three genes were significantly lower and fell below  ~ 15% (Fig. 7B). The fold changes for the *atpI*, *atpB* and *atpE* genes were  ~ 17-,  ~ 7- and  ~7-fold, respectively (Fig. 7B). These results are consistent with the interpretation that the L3S2P56 terminator introduced downstream of the promoter sequence very efficiently shuts-down further transcription. We conclude that we were able to engineer both polar and non-polar mutations in the *atp* locus. Moreover, the introduction of an artificial terminator sequence, without any deletion, phenocopies the growth defect induced in deletion mutants and, to a great extent, its changes in gene expression. Finally, our results suggest that under the conditions tested here, *atpIp* acts as the main promoter for *atpI*, *atpB*, and *atpE*, considering that a transcriptional terminator downstream of *atpIp* but upstream of two other described promoters (*atpBp1* and *atpBp2*) abolished their transcription [29].

### The inactivation or mutation of the a*tp* locus induces high intracellular ATP

**Fig. 8:**
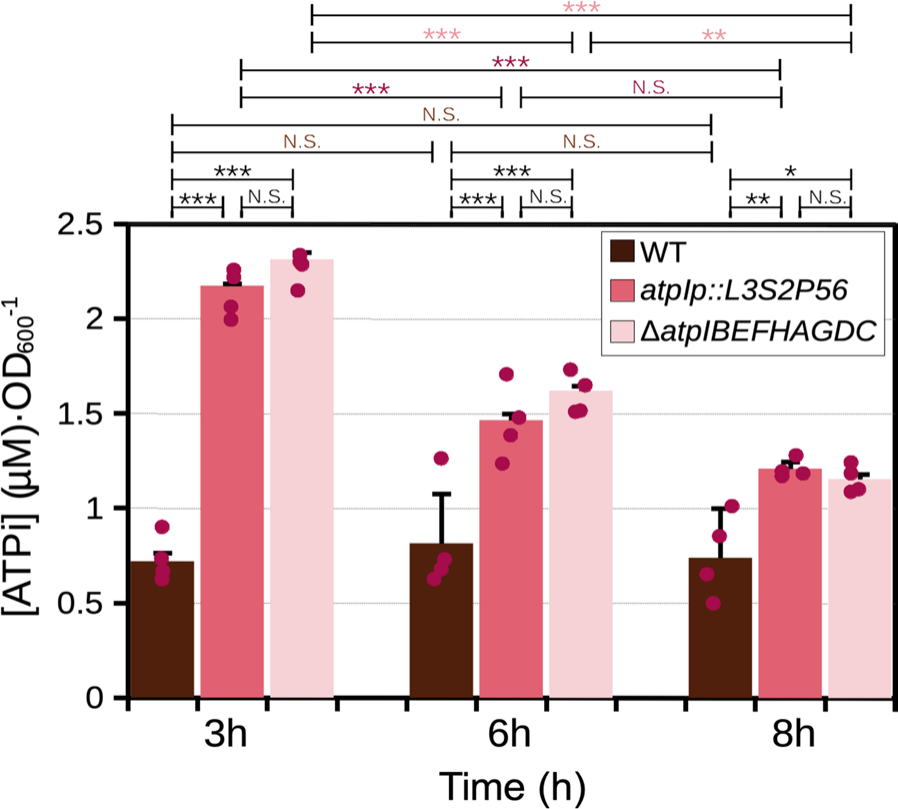
Effect of mutations in the *atpIBEFHAGDC* operon on intracellular ATP levels. [ATPi] values measured in whole cell extracts interpolated from [ATP] calibration curves at 3, 6 and 8 h of growth. The statistical analysis shows the results of a two-way ANOVA (panel B) followed by Tukey’s HSD multiple comparison tests. P values: *P* ≤ 0.001 (***), *P* ≤ 0.01 (**), *P* ≤ 0.05 (*), *NS* not significant

Next, we compared the Δ*atpIBEFHAGDC* mutant with the polar *atpIp::L3S2P56* insertion mutant to observe if they would phenotypically resemble each other. Therefore, to gain insight into the metabolic changes induced in these mutants, we measured the levels of intracellular ATP (ATPi). We measured luciferase activity in whole cell extracts [30] using a calibration curve to obtain the normalized µM ATP concentration at 3, 6 and 8 h of growth in minimal medium supplemented with glucose. Counter-intuitively, the Δ*atpIBEFHAGDC* showed significantly elevated levels of intracellular ATP at 3 and 6 h of growth. The maximum difference was at 3 h during exponential growth where this mutant had  ~ 3 times higher levels of ATPi (Fig. 8). However, at 8 h of growth, the difference was reduced to  ~ 1.5 times and below statistical significance. Interestingly, the *atpIp::L3S2P56* insertion mutant had levels of ATPi comparable to those of the Δ*atpIBEFHAGDC* deletion mutant (Fig. 8), showing the same range of significantly elevated ATPi during the exponential phase of growth. We conclude that the *atp* deletion mutant brings unexpected metabolic changes, which are consistent with previously published data [31]. Moreover, we conclude that the introduction of an artificial terminator sequence can be used as a general strategy to engineer the shutdown of complete polycistronic transcriptional units to manipulate the metabolism of *E. coli*.

## Discussion

In this study, we used artificial terminator sequences to modulate and shut down the transcription of the *fim* and *atp* operons. We took advantage of artificial sequences with extended secondary structures that improve their efficiency for transcriptional termination [11]. We did not know whether such secondary structures could impede the recombination process in the context of homology recombination with oligonucleotides used for CRISPR/Cas genome engineering. The predictable outcome from inserting these sequences in the *fim* and the *atp* operons is the premature transcriptional termination that would affect the expression of the downstream genes. Our results indeed showed up to a 43-fold reduction in the expression of the *fim* locus and up to a 17-fold reduction in the *atp* locus*.*

The efficiency of genome engineering in bacterial strains has been shown to depend on the efficiencies of gRNAs to direct Cas9 DNA cleavage and donor oligonucleotides [10]. In the case of the fim operon, using the same crRNA (crRNA1) to generate several mutations at the same target sequence, we observed that each mutation was produced with efficiencies ranging from 10 to 100%. However, the donor oligonucleotides varied in the sequence of homology arms in their secondary structure and length. As anticipated, our results suggest that these three parameters are important for bacterial genome engineering. Despite these differences, we could readily find mutants with inserted terminator sequences. Among the 582 terminator sequences characterised by Chen et al. [11], we first decided to use an artificial terminator sequence (L3S2P56) that was shown to have low recombination rates [11], which presumably would ensure more stability within the *E. coli* chromosome. Nevertheless, we did not know whether this would decrease the recombination mediated by homology arms upstream and downstream of the terminator sequence. Our results show that this sequence was quite easily engineered into the *E. coli* chromosome using the Cas12a-encoding plasmids designed by Yan et al. [22]. However, when compared with other termination sequences, the donor dsDNA oligonucleotides containing L3S2P56 showed low mutagenesis efficiency. Moreover, we observed some correlation between the stability of the potential secondary structure and the mutagenesis efficiency, suggesting that several parameters can be controlled during the design of donor oligonucleotides in the case of Cas12a-mediated polar mutagenesis strategies. Despite the lower recombination efficiency of donor sequences with L3S2P56, its termination strength allowed the complete shutdown of *atp* operon expression without the necessity to introduce any gene deletion. The plasmids used here in accordance with the description of Yan et al. [22] are easy to use and allow multiple rounds of mutagenesis similar to previously reported Cas9 approaches [10]. Cas12a also has the advantage that it processes its own crRNAs as described by Fonfara et al. [32], which we suggest will allow certain flexibility when dealing with organisms with poorly characterised promoter sequences. Altogether, this study shows that it is possible to shut down the expression of whole operons using artificial terminators. This approach could be complementary to other approaches that allow the transcriptional repression of multiple genes, such as the use of multiple gRNAs for transcriptional repression [33] or the deletion of large genomic stretches [34]. The use of Cas12a also facilitated genome engineering because its PAM sequence NTTT (AAAN in the minus strand) can be easily modified to introduce a stop codon. Indeed, here we showed that a single base substitution (*fimA*_*A106T*_) disrupted PAM recognition and allowed the selection of *fimA* mutant strains. In addition, the ability of Cas12a-mediated mutagenesis to generate relatively large modifications, such as the deletion of the *atpIBEFHAGDC* operon (~ 7 kb) in the *E. coli* chromosome without the need for any extra steps or additional fusion proteins, is notable [34].

The *fim* operon was an ideal study case because the phenotype is macroscopically observable and the characterisation of the *fimA* mutant constructs by yeast cell agglutination produced easy to interpret results. Moreover, the extent of fimbriation is directly correlated to the binary state of the phase variation switch involving the invertible *fimAp* DNA region [25]. Therefore, for every *fimA* transcript observed, the presence of a *fimI* transcript reflects the polarity of the mutants generated here. Indeed, insertion of a terminator sequence abolished the trans-complementation by a plasmid-encoded *fimA*^+^ gene. Less predictable phenotypes were obtained by measuring the relative expression of *fimI* to *fimA* under typical laboratory conditions. Three out of the four *fimA* mutant alleles studied here, both polar and non polar, had significantly reduced median levels of *fimA* transcripts, and all four mutant alleles produced a lower *fimI/fimA* ratio of transcripts. This included a single base substitution that introduced a stop codon *(fimA*_*A106T*_*).* Several possible explanations can be suggested to accommodate this fact. For example, it is known that ribosome binding protects mRNAs from RNase E degradation [35]. Therefore, one possible explanation would be that the premature stop codon within the *fimA*_*A106T*_ and deletion mutant (*ΔfimA*_*75*_) alleles induced premature release of ribosomes during translation [36, 37] and simultaneously exposed the *fimA* transcripts to degradation. Another more complicated explanation involves the stalling of ribosomes during translation, which may induce mRNA cleavage next to or at a stop codon [38]. An initial cleavage event could lead to further degradation by classical mRNA decay pathways. However, this could only make sense if the folding of the *fimA* mRNA would be compromised and stalling would be induced by the mutations designed here. This can also be put in the context of the recent discovery that the mRNA of individual genes within operons have a unique secondary structure [6]. Deletions smaller than a whole gene could affect the folding of an individual gene's mRNA within an operon. Another possibility would be that there would be positive feedback on type 1 fimbriae expression or functionality. Under the conditions that we assayed *fim* expression, which are rich medium at 37 °C, the off phase switching dominates the invertible *fimAp* DNA region [24]. Therefore, any kind of feedback would quickly result in *fimA* expression inhibition. Another possibility would be that the mutation introduced in this study interferes with differential stability within *fim* genes transcripts, which is an important element for other fimbrial systems (e.g., the *pap* fimbrial biogenesis operon in uropathogenic *E. coli*) [39]. One additional possibility is that the expression of a functional type 1 fimbriae system confers a growth advantage [39, 40], although this has only been proposed for static cultures. Alternatively, the peptide products of the stop codon mutants (~ 35 amino acids long and hydrophobic) could also be partially toxic and induce the selection of cells with the *fim* switch in the off state. However, this would not apply to the terminator insertion at position 1 (Δ*fimA*_*1*_*::L3S2P56*) mutant, for which there should not be any partial *fimA* peptide product. Further studies will be needed to clarify the role of the three stop codon mutants and the deletion mutant on the expression of *fimA.* In the case of the *atp* locus, the introduction of the complete deletion of the *atpB* gene also induced a small but statistically significant polar effect on the downstream gene *atpE.* One possibility applicable to the *fim* and *atp* loci explaining the reduced levels of a downstream gene upon mutation would be if any of the changes affects the occupancy of RNA polymerase in so-called occupancy domains, which have also been shown to be correlated to decreased co-expression of co-directionally expressed genes [41]. Altogether, our results show that even a single nucleotide substitution can cause subtle but measurable changes at the transcriptional level within an operon when measured under typical laboratory conditions. We can conclude here that it is difficult to predict a priori whether a particular mutagenesis strategy will introduce a measurable polar effect within a polycistronic transcriptional unit. It might also be interesting to consider the results presented here in the context of techniques that perform very efficient single nucleotide mutagenesis in bacteria by introducing premature stop codons such as Cas9 fused to a cytidine deaminase domain and, in general, any DNA base editors [42, 43].

The finding that markerless mutations causing shutdown of the whole *atp* locus transcription synthesise more ATPi during exponential growth suggests that these or similar mutants could be used to deliberately manipulate the levels of ATPi. Our results were consistent with previously published data showing that *E. coli* mutants with deletions within the *atp* locus had higher ATP-synthesis activities [31]. Interestingly, the growth rate during the exponential phase of all *atp* mutants tested here was comparable to that of the wild type strain. However, these strains would be at disadvantage during the early stationary phase, since they do not seem to reach the same mass of cells. Our results are consistent with previously published data showing lower mass production of an *E. coli* whole-operon *atp* deletion mutant [28]. Nevertheless, if a bio-production process is to occur during the exponential phase or independent of the mass of the cells but instead catalysed and dependent on the ATPi [44], these mutants could be interesting to test or combine with other mutations to manipulate *E. coli* or potentially other organisms.

On the one hand, the reduced number of terminator sequences used here represents a limitation of this study considering that terminator sequences can affect transcription in *E. coli* very differently depending on the genetic context and strength of the promoter [45]. Moreover, the termination strength measured by Chen et al. [11] might not be the same when tested in situ for other individual transcriptional units*.* On the other hand, the strategy presented here with individually-characterized terminator sequences could be very useful to fully isolate a transcriptional unit of interest when designing synthetic gene regulatory networks. This is important because it has been shown that transcriptional read-trough is very common in bacteria in co-directionally transcribed genes, even in adjacent genes from completely different operons [4, 41].

## Conclusions

In summary, in this study we show that Cas12a can be used to efficiently introduce markerless insertions in *E. coli* in one step and that insertions can include thermodynamically stable secondary structures such as transcriptional terminator sequences without excessively compromising the efficiency of the process. This approach allows the transcriptional shutdown of whole operons, as demonstrated for *fimAICDFGH* and *atpIBEFHAGDC* operons. This study also highlights the presence of potentially unwanted effects on mRNA levels when introducing all kinds of mutations. However, it is also possible that such an effect could represent an additional advantage to achieve polar mutagenesis to inactivate whole polycistronic genes. More importantly, the described strategy can be used to easily test the importance of entire operons for the physiology of *E. coli* or its effect in the design of novel or artificial biosynthetic pathways by manipulating its metabolism, as showcased by the increase in intracellular ATP in the markerless Δ*atpIBEFHAGDC* mutant.

## Methods

### Design of donor oligonucleotides for mutagenesis

To introduce polar and non polar mutations in the *fimAICDFGH* and *atpIBEFHAGDC* loci of *Escherichia coli* str. K-12 substr. MG1655 we designed two complementary oligonucleotides for each mutation to form dsDNA donor molecules for homologous recombination (Additional file 1: Table S2). The recombination of these oligonucleotides disrupts or deletes the binding site of the Cas12a-crRNA complex and permits the positive selection of mutant cells [9, 22]. To introduce a premature stop codon in the *fimA* gene, we designed dsDNA donor oligonucleotides in which a single bp or several bp changes disrupted the PAM sequence adjacent to the target of the designed *fimA* crRNA. To introduce a deletion in the *fimA* (Δ*fimA*) gene, *atpB* (Δ*atpB*), or *atpIBEFHAGDC* (Δ*atpIBEFHAGDC*) we designed dsDNA donor oligonucleotides containing homologous sequences for recombination upstream and downstream of the crRNA recognition site to delete 97 bp, 824 bp and 7033, respectively (Figs. 1 and 6). To engineer *E. coli* strains carrying polar mutations, the donor oligonucleotides had a terminator sequence between the homologous recombination sequences.

### Cas12a mutagenesis

To design Cas12a crRNA targeting *fimA*, *atpB* or *atpIp* with no off-targets in *E. coli* MG1655 [46], we used the webtool Chop-Chop (44). To introduce all the mutations designed in this study we used the plasmids designed by Yan et al. [22] and followed their protocol with some modifications. Cloning of crRNAs was performed by annealing forward and reverse oligonucleotides at 95 °C and ramping the temperature down at 2.5 °C/min. The oligonucleotides were then phosphorylated using polynucleotide kinase (ThermoFisher). The plasmid pAC-crRNAred [22] was digested with BsaI-HF (New England Biolabs), dephosphorylated with FastAP (ThermoFisher) and ligated to the annealed oligonucleotides. Donor oligonucleotides were treated in the same way as crRNA oligonucleotides and their annealing was verified as suggested by König et al. [47]*.* The night before the experiment we grew *E. coli* MG1655 carrying plasmid pKD46-Cas12a at 30 °C starting from a glycerol stock in liquid LB. The day of the experiment, 100 μl of overnight culture was spread on a new agar plate containing 0.2% arabinose and 4 h later electrocompetent cells were prepared using the “rapid” protocol [48]. These cells were co-transformed by plasmids carrying one crRNA and an annealed pair of donor oligonucleotides. As a control transformation we used a crRNA targeting the gene coding for green fluorescent protein (GFP). Colony PCR analysis was then performed on transformants for genotyping. Each colony was resuspended in 20 μl deionized water and boiled for 5 min. One microlitre of this sample was used as template for PCR. Agarose gel electrophoresis or Sanger sequencing was performed to determine the number of validated mutants. Alternatively, for *fimA* mutants using crRNA1, crRNA2 and crRNA3, *E. coli* colonies were replica-plated after co-transformation and tested for yeast cell agglutination test (see below) to see if they displayed a Fim negative phenotype before colony PCR test and sequencing. After mutagenesis was confirmed by sequencing, the strains were cured from the plasmids following the recommendations of Yan et al. [22]. We observed that this process had a 100% efficiency by growing the cells at 37 °C in 5% sucrose in very rich medium such as YT2X. Glycerol stocks were then prepared for each clone. The structure and free energy of the terminator sequences were calculated with the Vienna websuite [27] and visualised with Forna [49]. Altogether, from plating the *E. coli* strain carrying the pKD46-Cas12a plasmid to the genotyping of possible mutants, it takes 3 days.

### Agglutination assay

To observe yeast cell agglutination, wild type or mutant MG1655 was grown overnight for 16 h on Luria agar plates as described previously [50]. A 1 μl plastic loop was used to take a colony from the plate and mixed with 200 μl of a *Saccharomyces cerevisiae* suspension at OD_600_ 5 prepared from refrigerated baker’s yeast (Jästbolaget). The loop was used to gently mix the two cell populations, and the appearance of white flocculated aggregates could be observed for the wild type strain in less than 30 s. *Shigella flexneri* 5A M90T (Additional file 1: Table S1) *was* used as a negative control [51]. To perform this assay from static liquid cultures, 10 μl from strains grown overnight in static broth (20 ml tryptic soy broth) were mixed with 200 μl of a *Saccharomyces cerevisiae* suspension at OD_600_ 5. In the case of the wild type *E. coli* strain expressing the plasmid-encoded *fimA*^+^ allele, agglutination took approximately 2–5 min under these conditions. As a control to verify that the agglutination was mannose-specific, 10 μl of a 3% (w/v) solution of α-methyl-mannoside was added to a 10 μl suspension of wild type *E. coli* and preincubated for 1 min before testing for yeast cell agglutination. Figure 2A–C was created with BioRender.com.

### RNA isolation

To isolate RNA, we used the modified method described by Blomberg et al. [52] with one further modification for samples to measure *fim* expression. Namely, we grew cells in the same conditions as for agglutination assays (overnight on Luria agar plates) as a lawn. The equivalent to half a plate was harvested and dissolved immediately in DEPC treated water with 10% SDS, 20 mM sodium acetate pH 4.8 and 10 mM EDTA and flash frozen in liquid nitrogen. For cells prepared to measure *atp* expression, cells were grown in minimal medium (see below) for 3 h and flash frozen. Frozen cells were thawed and lysed by incubating the samples for 5 min at 65 °C. Total RNA was extracted by the hot-phenol method [52]. Residual DNA was digested by DNase I (ThermoFisher; 1 U/μg RNA, 60 min, 37 °C) in the presence of RNase inhibitor (Ribolock, ThermoFisher Scientific; 0.1 U/μl) followed by a cleaning step using phenol/chloroform/isoamyl alcohol (25:24:1) and precipitation with ethanol, 0.1 M sodium acetate pH 5.5 and 20 μg of glycogen (ThermoFisher). Removal of residual DNA was verified by PCR using *fimA* specific oligonucleotides. Complementary DNAs (cDNAs) were prepared from each RNA sample using the RevertAid kit (ThermoFisher) according to the manufacturer’s instructions. As a control for DNA contamination in the qPCR test, we prepared RNA a cDNA reaction tube without reverse transcriptase (-RT sample) for each purified RNA.

### qPCR analysis

To perform qPCR we used SYBR Green master mix (ThermoFisher) according to the manufacturer's instructions. To analyse the expression of *fimA*, *fimI*, *atpI*, *atpB*, *atpE, hcaT* and *cysG*, we designed specific oligonucleotides using primer BLAST (Additional file 1: Table S2). We performed qPCR in an iCycler iQ5 (Bio-Rad) with cDNAs generated from bacteria grown on plates or minimal AB medium and as a control of DNA contamination we produced a sample without reverse transcriptase (-RT) using the same amount of RNA. We verified that for each qPCR experiment we did not find any detectable signal in the -RT sample. Denaturation curves were also acquired to verify the presence of a single product. We performed these controls for every single biological replicate. To verify the linearity of each primer we diluted the cDNA obtained from wild type *E. coli*, which always contained the highest amounts of *fimA and fimI* and verified that 10 times dilutions steps had a slope close to − 3.3. To analyse qPCR data, we used the iQ5 optical system software (Bio-Rad) and obtained the ΔCt from the suggested values of the software. We manually verified in any case that changes in the threshold did not affect or bias the results. Each measurement was quantified using the following formula: ΔCt = 2^(ct1 − ct2), where ct is the threshold crossing value at which the SYBR green fluorescent signal appears. In the case of the *fimI* to *fimA* ratios reported in Fig. 4A, we simply report the ΔCt values without any transformation. Although Ct values varied across experiments, the *fimI* to *fimA* ΔCt was, throughout this study, consistent across experiments within one order of magnitude. To normalize the data of *fimA* and *fimI* against housekeeping genes we obtained the ΔCt of each sample against the ct value of *cysG* and *hcaT*, and the average of the two values was calculated. The average ΔCt values of each sample were normalized against the average ΔCt of the technical replicates of the wild type strain to obtain a percentage. The ratio of the *fimI* to *fimA* expression was obtained by dividing their normalized ΔCt values and expressed as a percentage.

### AFM imaging

To prepare AFM samples, punch-holed 1 cm pieces of muscovite mica were immobilized on metal supports using double-sided tape. The static cultures were diluted ten times in ultrapure milliQ water. A drop of 100 μl from the diluted bacterial suspension was then placed on the mica right after cleaving it with laboratory tape. The suspension was incubated for 20 min to allow adhesion of bacteria and the liquid was exchanged three times with ultrapure milli Q water. The samples were placed in the chemical hood and left to dry for a minimum of 5 min. The presence of bacteria on the mica was verified using an optical system mounted on the AFM and a random area was selected for imaging. AFM imaging was performed in a MultiMode8 instrument (Bruker) in force tapping mode using the ScanAsyst software at a rate of 1 Hz and ScanAsyst tips with a nominal spring constant of 0.4 N/m. The images shown in Fig. 3 A, B and Additional file 1: Fig. S3 depict the error signal generated by the piezo (J scanner).

### Bacterial growth curves

Bacterial cells were grown overnight in AB minimal medium [53] supplemented with 0.2% glucose, thiamine (10 µg/ml) and uracil (25 µg/ml) [54]. The next day, cultures were diluted to an OD of 0.05, transferred to a 96 well plate and incubated at 37 °C with shaking inside a plate reader (BIOTEK). The absorbance at 600 nm was recorded every 15 min for 15 h.

### Intracellular ATP (ATPi) measurements

The method was modified from Yang et al. [30]. Bacterial cells were grown overnight in AB minimal medium as above. The next day, cultures were diluted to an OD of 0.05 and incubated in a rotary shaker at 37 °C. After 3, 6 and 8 h of growth, 1.5 ml samples were taken from the culture and centrifuged at 12 000 RPM for 2 min at RT. The pellet was immediately resuspended in 1 ml of deionized water at near boiling temperature. The samples were then centrifuged at 20,000 RPM for 5 min to remove cell debris. The concentration of ATP was immediately measured in samples from the super-natant using a commercial luciferase kit (ATP determination kit PRO. Biaffin, GmbH). To prepare a standard cell curve, we measured [ATP] at each time point using the same kit in a range of 0–5 µM diluted in water from an ATP stock (25 mM, ThermoFisher).

### Statistical analysis

Statistical analysis was performed in R and GraphPad Prism. Linear regression statistical analysis was performed in R to obtain the p value of the R^2^. The 95% confidence interval of the forecasted ŷ in the linear regression was calculated as ŷ ± *t*_*inv*_*·SE, where t*_*inv*_ is the *t* inverse distribution value for α_0.05/2_ with n-2 degrees of freedom and SE is the standard error.

## Supplementary Information

**Additional file 1: Table S1.** Strains used in this study. **Table S2.** Oligos used. **Figure S1.** Structure of terminator sequences. A selected set of terminator transcript sequences from the compilation of 582 terminator sequences characterised by Chen et al. by their termination efficiency (Te) [3].The calculated free energy of the secondary structure is shown below each transcript sequence. **Figure S2.** Efficiency of *fimA* mutagenesis with Cas12a. **A** 10/10 *E.* *coli* colonies isolated after cotransformation with crRNA1 and the donor oligonucleotide carrying homology arms to introduce a 97 bp *fimA* deletion tested positive (lanes 1–10) for a mutation as detected by colony PCR using primers F1 and R1. **B** 3/10 *E.* *coli* colonies isolated after co-transformation with crRNA1 and the donor oligonucleotide carrying homology arms to insert a 57 bp terminator beside the 97 bp *fimA* deletion at position 75 of the *fimA* (Δ*fimA*_75_*::L3S2P56*) gene tested positive (lanes 1, 5 and 7 marked with *) as detected by colony PCR using primers F1 and R1. **C** 1/10 *E.* *coli* colonies isolated after co-transformation with crRNA1 and the donor oligonucleotide carrying homology arms to insert a 57 bp terminator sequence beside the 172 bp *fimA* deletion at position 1 of the *fimA* (Δ*fimA*_1_*::L3S2P56*) gene tested positive (lane 5, marked with *) as detected by colony PCR using primers F2 and R2. Lane 11 shows a PCR product on WT *E.* *coli* DNA template as detected with primers F1 and R1. Lanes labeled as L were loaded with a double-stranded DNA ladder containing fragments of different lengths in base pairs (bp). **Figure S3.** Atomic force microscopy (AFM) of bacteria grown in static cultures. **A** AFM imaging of *E.* *coli* cells with the *ΔfimA*_*75*_ allele carrying the empty vector pSU19. **A** *E.* *coli* cell showing no type 1 fimbriae is shown in the inset. **B** AFM imaging of the *ΔfimA*_*75*_ mutant *E.* *coli* carrying a plasmid containing a *fimA*^*+*^ allele for transcomplementation. **A** *E.* *coli* cell showing type 1 fimbriae is shown in the inset. Scale bars: **A**, **B** 4 μm. **A**, **B** insets 2 μm. **Figure S4.** Thermodynamic properties of terminator sequences. Correlation of the efficiency of mutagenesis and the following thermodynamic parameters: **A** free energy for the closure of the hairpin loop (∆GL). **B** free energy of the hairpin folding (ΔGH). **C** Free energy of the extended hairpin (∆GA). **D** Free energy of the base of the stem (∆GB). **E** Free energy of the U-tract (∆GU), **F** length (bp).

## Data Availability

The minimal dataset necessary to interpret, replicate and build upon the findings reported in the article are found in the supplementary material file. Raw data are available from the corresponding author upon reasonable request.

## References

[1] Fondi M, Emiliani G, Fani R (2009). Origin and evolution of operons and metabolic pathways. Res Microbiol.

[2] Cao H, Ma Q, Chen X, Xu Y (2019). DOOR: a prokaryotic operon database for genome analyses and functional inference. Brief Bioinform.

[3] Che D, Li G, Mao F, Wu H, Xu Y (2006). Detecting uber-operons in prokaryotic genomes. Nucleic Acids Res.

[4] Junier I, Rivoire O (2016). Conserved units of co-expression in bacterial genomes: an evolutionary insight into transcriptional regulation. PLoS ONE.

[5] Lathe WC, Snel B, Bork P, Lathe WC, Snel B, Bork P (2000). Gene context conservation of a higher order than operons. Trends Biochem Sci.

[6] Burkhardt DH, Rouskin S, Zhang Y, Li G-W, Weissman JS, Gross CA (2017). Operon mRNAs are organized into ORF-centric structures that predict translation efficiency. Elife.

[7] Goodson JR, Winkler WC (2018). Processive antitermination. Microbiol Spectr.

[8] Merino E, Yanofsky C (2005). Transcription attenuation: a highly conserved regulatory strategy used by bacteria. Trends Genet.

[9] Jiang W, Bikard D, Cox D, Zhang F, Marraffini LA (2013). RNA-guided editing of bacterial genomes using CRISPR-Cas systems. Nat Biotechnol.

[10] Zerbini F, Zanella I, Fraccascia D, König E, Irene C, Frattini LF (2017). Large scale validation of an efficient CRISPR/Cas-based multi gene editing protocol in *Escherichia coli*. Microb Cell Fact.

[11] Chen Y-J, Liu P, Nielsen AA, Brophy JA, Clancy K, Peterson T (2013). Characterization of 582 natural and synthetic terminators and quantification of their design constraints. Nat Methods.

[12] Duguid JP, Smith IW, Dempster G, Edmunds PN (1955). Non-flagellar filamentous appendages (“fimbriae”) and haemagglutinating activity in *Bacterium coli*. J Pathol Bacteriol.

[13] Jones CH, Pinkner JS, Roth R, Heuser J, Nicholes AV, Abraham SN (1995). FimH adhesin of type 1 pili is assembled into a fibrillar tip structure in the *Enterobacteriaceae*. Proc Natl Acad Sci.

[14] Krogfelt KA, Bergmans H, Klemm P (1990). Direct evidence that the FimH protein is the mannose-specific adhesin of *Escherichia coli* type 1 fimbriae. Infect Immun.

[15] Collier WA, De Miranda JC (1955). Bacterial hemagglutination. III. Mannose inhibition of E*. coli *hemagglutination. Antonie Van Leeuwenhoek.

[16] Hultgren SJ, Porter TN, Schaeffer AJ, Duncan JL (1985). Role of type 1 pili and effects of phase variation on lower urinary tract infections produced by *Escherichia coli*. Infect Immun.

[17] Wright KJ, Seed PC, Hultgren SJ (2007). Development of intracellular bacterial communities of uropathogenic *Escherichia coli* depends on type 1 pili. Cell Microbiol.

[18] Spaulding CN, Klein RD, Ruer S, Kau AL, Schreiber HL, Cusumano ZT (2017). Selective depletion of uropathogenic *E.*
*coli* from the gut by a FimH antagonist. Nature.

[19] Korea C-G, Badouraly R, Prevost M-C, Ghigo J-M, Beloin C (2010). *Escherichia coli* K-12 possesses multiple cryptic but functional chaperone–usher fimbriae with distinct surface specificities. Environ Microbiol.

[20] Jones HM, Brajkovich CM, Gunsalus RP (1983). In vivo 5ʹ terminus and length of the mRNA for the proton-translocating ATPase (unc) operon of *Escherichia*
*coli*. J Bacteriol.

[21] Hara KY, Kondo A (2015). ATP regulation inbioproduction. Microb Cell Fact.

[22] Yan M-Y, Yan H-Q, Ren G-X, Zhao J-P, Guo X-P, Sun Y-C (2017). CRISPR-Cas12a-assisted recombineering in bacteria. Appl Environ Microbiol.

[23] Bravo V, Puhar A, Sansonetti P, Parsot C, Toro CS (2015). Distinct mutations led to inactivation of type 1 fimbriae expression in *Shigella *spp.. PLoS ONE.

[24] Blomfield IC, McClain MS, Princ JA, Calie PJ, Eisenstein BI (1991). Eisenstein BI. Type 1 fimbriation and *fimE* mutants of *Escherichia coli* K-12. J Bacteriol.

[25] Abraham JM, Freitag CS, Clements JR, Eisenstein BI (1985). An invertible element of DNA controls phase variation of type 1 fimbriae of *Escherichia coli*. PNAS.

[26] Zhou K, Zhou L, Lim QE, Zou R, Stephanopoulos G, Too H-P (2011). Novel reference genes for quantifying transcriptional responses of *Escherichia coli* to protein overexpression by quantitative PCR. BMC Mol Biol.

[27] Gruber AR, Lorenz R, Bernhart SH, Neuböck R, Hofacker IL (2008). The Vienna RNA websuite. Nucleic Acids Res.

[28] Jensen PR, Michelsen OLE (1992). Carbon and energy metabolism of *atp* mutants of *Escherichia coli*. J Bacteriol.

[29] Nielsen J, Jørgensen BB, van Meyenburg KV, Hansen FG (1984). The promoters of the *atp* operon of *Escherichia coli* K12. Mol Gen Genet.

[30] Yang N-C, Ho W-M, Chen Y-H, Hu M-L (2002). A convenient one-step extraction of cellular ATP using boiling water for the luciferin–luciferase assay of ATP. Anal Biochem.

[31] Hara KY, Mori H (2006). An efficient method for quantitative determination of cellular ATP synthetic activity. J Biomol Screen.

[32] Fonfara I, Richter H, Bratovič M, Le Rhun A, Charpentier E (2016). The CRISPR-associated DNA-cleaving enzyme Cpf1 also processes precursor CRISPR RNA. Nature.

[33] Cress BF, Toparlak ÖD, Guleria S, Lebovich M, Stieglitz JT, Englaender JA (2015). CRISPathBrick: modular combinatorial assembly of type II-A CRISPR arrays for dCas9-mediated multiplex transcriptional repression in *E.*
*coli*. ACS Synth Biol.

[34] Su T, Liu F, Gu P, Jin H, Chang Y, Wang Q (2016). A CRISPR-Cas9 assisted non-homologous end-joining strategy for one-step engineering of bacterial genome. Sci Rep.

[35] Richards J, Belasco JG (2019). Obstacles to scanning by RNase E govern bacterial mRNA lifetimes by hindering access to distal cleavage sites. Mol Cell.

[36] Laurberg M, Asahara H, Korostelev A, Zhu J, Trakhanov S, Noller HF (2008). Structural basis for translation termination on the 70S ribosome. Nature.

[37] Scolnick E, Tompkins R, Caskey T, Nirenberg M (1968). Release factors differing in specificity for terminator codons. Proc Natl Acad Sci USA.

[38] Hayes CS, Sauer RT (2003). Cleavage of the A site mRNA codon during ribosome pausing provides a mechanism for translational quality control. Mol Cell.

[39] Båga M, Göransson M, Normark S, Uhlin BE (1988). Processed mRNA with differential stability in the regulation of *E.*
*coli* pilin gene expression. Cell.

[40] Old DC, Duguid JP (1970). Selective outgrowth of fimbriate bacteria in static liquid medium. J Bacteriol.

[41] Junier I, Unal EB, Yus E, Lloréns-Rico V, Serrano L (2016). Insights into the mechanisms of basal coordination of transcription using a genome-reduced bacterium. Cell Syst.

[42] Nishida K, Arazoe T, Yachie N, Banno S, Kakimoto M, Tabata M (2016). Targeted nucleotide editing using hybrid prokaryotic and vertebrate adaptive immune systems. Science.

[43] Hess GT, Tycko J, Yao D, Bassik MC (2017). Methods and applications of CRISPR-mediated base editing in eukaryotic genomes. Mol Cell.

[44] Tao S, Qian Y, Wang X, Cao W, Ma W, Chen K (2018). Regulation of ATP levels in *Escherichia*
*coli* using CRISPR interference for enhanced pinocembrin production. Microb Cell Fact.

[45] Lin M-T, Wang C-Y, Xie H-J, Cheung CHY, Hsieh C-H, Juan H-F (2016). Novel utilization of terminators in the design of biologically adjustable synthetic filters. ACS Synth Biol.

[46] Guyer MS, Reed RR, Steitz JA, Low KB (1981). Identification of a sex-factor-affinity site in *E.**coli* as γδ. Cold Spring Harbor symposia on quantitative biology.

[47] König E, Zerbini F, Zanella I, Fraccascia D, Grandi G (2018). Multiple stepwise gene knockout using CRISPR/Cas9 in *Escherichia coli*. Bio-Protoc.

[48] Gonzales MF, Brooks T, Pukatzki SU, Provenzano D (2013). Rapid protocol for preparation of electrocompetent *Escherichia coli* and *Vibrio cholerae*. JoVE.

[49] Kerpedjiev P, Hammer S, Hofacker IL (2015). Forna (force-directed RNA): simple and effective online RNA secondary structure diagrams. Bioinformatics.

[50] Müller CM, Åberg A, Straseviçiene J, Emödy L, Uhlin BE, Balsalobre C (2009). Type 1 fimbriae, a colonization factor of uropathogenic *Escherichia coli*, are controlled by the metabolic sensor CRP-cAMP. PLoS Pathog.

[51] Sansonetti PJ, Kopecko DJ, Formal SB (1982). Involvement of a plasmid in the invasive ability of *Shigella flexneri*. Infect Immun.

[52] Blomberg P, Wagner EG, Nordström K (1990). Control of replication of plasmid R1: the duplex between the antisense RNA, CopA, and its target, CopT, is processed specifically in vivo and in vitro by RNase III. EMBO J.

[53] Clark DJ, Maaløe O (1967). DNA replication and the division cycle in *Escherichia coli*. J Mol Biol.

[54] Jensen KF (1993). The *Escherichia coli* K-12" wild types" W3110 and MG1655 have an rph frameshift mutation that leads to pyrimidine starvation due to low pyrE expression levels. J Bacteriol.

