# Supplementary material for: Polar mutagenesis of polycistronic bacterial transcriptional units using Cas12a
Source: Microb Cell Fact. 2022 Jul 13;21:139. doi: 10.1186/s12934-022-01844-y (PMC9277811; doi:10.1186/s12934-022-01844-y)
Supplement: Supplementary file 1 — Additional file 1: Table S1. Strains used in this study. Table S2. Oligos used. Figure S1. Structure of terminator sequences. A selected set of terminator transcript sequences from the compilation of 582 terminator sequences characterised by Chen et al. by their termination efficiency (Te) [3].The calculated free energy of the secondary structure is shown below each transcript sequence. Figure S2. Efficiency of fimA mutagenesis with Cas12a. A 10/10 E. coli colonies isolated after cotransformation with crRNA1 and the donor oligonucleotide carrying homology arms to introduce a 97 bp fimA deletion tested positive (lanes 1–10) for a mutation as detected by colony PCR using primers F1 and R1. B 3/10 E. coli colonies isolated after co-transformation with crRNA1 and the donor oligonucleotide carrying homology arms to insert a 57 bp terminator beside the 97 bp fimA deletion at position 75 of the fimA (ΔfimA75::L3S2P56) gene tested positive (lanes 1, 5 and 7 marked with *) as detected by colony PCR using primers F1 and R1. C 1/10 E. coli colonies isolated after co-transformation with crRNA1 and the donor oligonucleotide carrying homology arms to insert a 57 bp terminator sequence beside the 172 bp fimA deletion at position 1 of the fimA (ΔfimA1::L3S2P56) gene tested positive (lane 5, marked with *) as detected by colony PCR using primers F2 and R2. Lane 11 shows a PCR product on WT E. coli DNA template as detected with primers F1 and R1. Lanes labeled as L were loaded with a double-stranded DNA ladder containing fragments of different lengths in base pairs (bp). Figure S3. Atomic force microscopy (AFM) of bacteria grown in static cultures. A AFM imaging of E. coli cells with the ΔfimA75 allele carrying the empty vector pSU19. A E. coli cell showing no type 1 fimbriae is shown in the inset. B AFM imaging of the ΔfimA75 mutant E. coli carrying a plasmid containing a fimA+ allele for transcomplementation. A E. coli cell showing type 1 fimbriae is shown in the inset. Scale bars: [file 12934_2022_1844_MOESM1_ESM.pdf]

Polar mutagenesis of polycistronic bacterial transcriptional units using Cas12a

Antoine Graffeuil[1,3,¶], Julio Guerrero-Castro[1,3¶], Aster Assefa[1,3], Bernt Eric Uhlin[1,2,3] and David A. Cisneros[1,3*]

[1]Department of Molecular Biology, Umeå University, Umeå, Sweden.

[2]The Laboratory for Molecular Infection Medicine Sweden (MIMS), Umeå, Sweden.

[3]Umeå Centre for Microbial Research (UCMR), Umeå, Sweden.

*Corresponding author: david.cisneros@umu.se

¶These authors contributed equally.

Supplementary material

Table 1. Strains used in this study

Strain	Reference
Escherichia coli K-12 substr. MG1655	[1]
Shigella flexneri 5A M90T	[2]
Escherichia coli fimA$_{A106T}$	This study
Escherichia coli fimA$_{T103A,T104A}$	This study
Escherichia coli fimA$_{A158T,A159T,A160T,C161A,C162A}$	This study
Escherichia coli ΔfimA$_{75}$	This study
Escherichia coli ΔfimA$_{75}$*::L3S2P56*	This study
Escherichia coli ΔfimA$_{1}$*::L3S2P56*	This study
ΔatpIBEFHAGDC	This study
ΔatpB	This study
atpB::L3S3P41	This study
atpIp::L3S2P56	This study

Table 1. Strains used in this study

Table 2. Oligos used

Name (description)	Sequence
fimA genotyping (Figure 1 primer F1)	GGCAATCGTTGTTCTGTCGG
fimA genotyping (Figure 1 primer R1)	GAAGGTCGCATCCGCATTAG
ΔfimA1::L3S2P56 for terminator genotyping (Figure 1 primer F2)	CTGGAACCGTAACGCAGACT
ΔfimA1::L3S2P56 for terminator genotyping (Figure 1 primer R2)	AGGCAACAGCGGCTTTAGAT
ΔatpIBEFHAGDG genotyping (Figure 1 primer F3)	aatcggcgctaagaaccatc
ΔatpIBEFHAGDG genotyping (Figure 1 primer R3)	ggatctatgtgaacgctattcagg
ΔatpB genotyping (Figure 1 primer R4)	CAGCAGAGGAATCAGATCAGG
ΔatpIp::L3S2P56 genotyping (Figure 1 primer R5)	GCAATCCACTTGCTATCACC
fimA+ cloning pSU19 HindIII EcoRI forward	tacgCCAAGCTTTGAAAGGAAAGCAGCATGAAAATTAAAAC
fimA+ cloning pSU19 HindIII EcoRI reverse	gccagtGAATTCTTATTGATACTGAACCTTGAAGGTCG
crRNA GFP BsaI control Fw to clone target sequence	tagatCGTCGCCGTCCAGCTCGACCAGGA
crRNA GFP BsaI control Rv to clone target sequence	agacTCCTGGTCGAGCTGGACGGCGACGa
crRNA1 fimA BsaI Fw to clone target sequence	tagatAAGTGAACGGTCCCACCATTAACC
crRNA1 fimA BsaI Rv to clone target sequence	agacGGTTAATGGTGGGACCGTTCACTTa
crRNA2 fimA BsaI Fw to clone target sequence	tagatAAGGGGAAGTTGTTAACGCCGCTT
crRNA2 fimA BsaI Rv to clone target sequence	agacAAGCGGCGTTAACAACTTCCCCTTa
crRNA3 fimA BsaI Fw to clone target sequence	tagatATCAACAGAGCCTGCATCAACTGC
crRNA3 fimA BsaI Rv to clone target sequence	agacGCAGTTGATGCAGGCTCTGTTGATa
crRNA4 atpB BsaI Fw to clone target sequence	tagatTGAAGCCATGATGCCTTTTACCCT
crRNA4 atpB BsaI Rv to clone target sequence	agacAGGGTAAAAGGCATCATGGCTTCAa
crRNA5 atpIp BsaI Fw to clone target sequence	tagatTACGACACGCGGCATACCTCGAAG
crRNA5 atpIp BsaI Rv to clone target sequence	agacCTTCGAGGTATGCCGCGTGTCGTAa
qPCR fimA Fw 90 bp recognizes all mutants	ATCTTTTGGGGGAAAACTGTGC
qPCR fimA Rv 90 bp recognizes all mutants	ATTTCTAAATCGACATGGGCAGT
qPCR fimI Fwd 233pb	TGCTGCCAATGTTTGCTCTG
qPCR fimI Rev 233pb	TTCACTCACCACCGTGCTAC
qPCR cysG Fwd 237 pb	CGCTCGCTTAACGGTGAATG
qPCR cysG Rev 237 pb	GGCATAATAAAGCTGGCGGC
qPCR hcaT Fwd 236 pb	TGTAGTGCACGCGATATGCT
qPCR hcaT Rev 236 pb	ATGATAGCGATACTGCCGCC
ΔfimA deletion donor oligonucleotide Fw	GGCTCTGTCCCTCAGTTCTACAGCGGCTCTGGCCGCTGCCtaaGACAGGTTCGTAC CGCATCGCTGGCACAGGAAGGAGCAAC
ΔfimA deletion donor oligonucleotide Rv	GTTGCTCCTTCCTGTGCCAGCGATGCGGTACGAACCTGTCttaGGCAGCGGCCAGA GCCGCTGTAGAACTGAGGGACAGAGCC
fimAA106T donor oligonucleotide Fw	TGCCACGACGGTTAATGGTGGGACCGTTCACTTTTAAGGGGAAGTTGTTAACGCCG CTTGCGCAGTTGAT
fimAA106T donor oligonucleotide Rv	ATCAACTGCGCAAGCGGCGTTAACAACTTCCCCTTAAAAGTGAACGGTCCCACCAT TAACCGTCGTGGCA
fimAT103A-T104A donor oligonucleotide Fw with crRNA2	CTGCCACGACGGTTAATGGTGGGACCGTTCACTAAAAAGGGGAAGTTGTTAACGCC GCTTGCGCAGTTGA
fimAT103A-T104A donor oligonucleotide Rv with crRNA2	TCAACTGCGCAAGCGGCGTTAACAACTTCCCCTTTTTAGTGAACGGTCCCACCATT AACCGTCGTGGCAG
fimAA158T-A159T-A160T-C161A-C162A) donor oligonucleotid Fw with crRNA3	GCCGCTTGCGCAGTTGATGCAGGCTCTGTTGATCTTTAAGTTCAGTTAGGACAGGT TCGTACCGCATCGC
fimAA158T-A159T-A160T-C161A-C162A) donor oligonucleotid Rv with crRNA3	GCGATGCGGTACGAACCTGTCCTAACTGAACTTAAAGATCAACAGAGCCTGCATCA ACTGCGCAAGCGGC
ΔfimA1::L3S2P56 donor oligonucleotide Fw	TTAGAAATAGTTTTTTGAAAGGAAAGCAGCCTCGGTACCAAATTTTCGAAAAAAGAC GCTGAAAAGCGTCTTTTTTCGTTTTGGTCCGACAGGTTCGTACCGCATCGCTGGCA CAGG
ΔfimA1::L3S2P56 donor oligonucleotide Rv	CCTGTGCCAGCGATGCGGTACGAACCTGTCGGACCAAAACGAAAAAAGACGCTTTT CAGCGTCTTTTTTCGAAAATTTGGTACCGAGGCTGCTTTCCTTTCAAAAAACTATTTC TAA
ΔfimA75::L3S2P56 donor oligonucleotide Fw	CCTGTGCCAGCGATGCGGTACGAACCTGTCGGACCAAAACGAAAAAAGACGCTTTT CAGCGTCTTTTTTCGAAAATTTGGTACCGAGGGCAGCGGCCAGAGCCGCTGTAGAA CTGAG
ΔfimA75::L3S2P56 donor oligonucleotide Rv	CCTGTGCCAGCGATGCGGTACGAACCTGTCGGACCAAAACGAAAAAAGACGCTTTT CAGCGTCTTTTTTCGAAAATTTGGTACCGAGGGCAGCGGCCAGAGCCGCTGTAGAA CTGAG

Table 2 (continues)

ΔfimA75::L3S3P41 donor oligonucleotide Fw	CTCAGTTCTACAGCGGCTCTGGCCGCTGCCAAAAAAAAAAAAACACCCTAACGGGTG TTTTTTTTTTTTTGGTCTCCCGACAGGTTCGTACCGCATCGCTGGCACAGG
ΔfimA75::L3S3P41 donor oligonucleotide Rv	CCTGTGCCAGCGATGCGGTACGAACCTGTCGGGAGACCAAAAAAAAAAAAAACACC CGTTAGGGTGTTTTTTTTTTTTTGGCAGCGGCCAGAGCCGCTGTAGAACTGAG
ΔfimA75::L3S3P21 donor oligonucleotide Fw	CTCAGTTCTACAGCGGCTCTGGCCGCTGCCCCAATTATTGAAGGCCTCCCTAACGG GGGGCCTTTTTTTGTTTCTGGTCTCCCGACAGGTTCGTACCGCATCGCTGGCACAG G
ΔfimA75::L3S3P21 donor oligonucleotide Rv	CCTGTGCCAGCGATGCGGTACGAACCTGTCGGGAGACCAGAAACAAAAAAAGGCC CCCCGTTAGGGAGGCCTTCAATAATTGGGGCAGCGGCCAGAGCCGCTGTAGAACT GAG
ΔfimA75::L3S3P56 donor oligonucleotide Fw	CTCAGTTCTACAGCGGCTCTGGCCGCTGCCTTTTCGAAAAAACACCCTAACGGGTG TTTTTTTGTTTCTGGTCTCCCGACAGGTTCGTACCGCATCGCTGGCACAGG
ΔfimA75::L3S3P56 donor oligonucleotide Rv	CCTGTGCCAGCGATGCGGTACGAACCTGTCGGGAGACCAGAAACAAAAAAACACC CGTTAGGGTGTTTTTTCGAAAAGGCAGCGGCCAGAGCCGCTGTAGAACTGAG
ΔfimA75::Bba_B0062 donor oligonucleotide Fw	CTCAGTTCTACAGCGGCTCTGGCCGCTGCCcagataaaaaaaatccttagctttcgctaaggatgattt ctGACAGGTTCGTACCGCATCGCTGGCACAGG
ΔfimA75::Bba_B0062 donor oligonucleotide Rv	CCTGTGCCAGCGATGCGGTACGAACCTGTCagaaatcatccttagcgaaagctaaggattttttttatct gGGCAGCGGCCAGAGCCGCTGTAGAACTGAG
ΔfimA75::L2U1H10 donor oligonucleotide Fw	CTCAGTTCTACAGCGGCTCTGGCCGCTGCCGGGCGGTCAGATGATCGCCCTTTTTT TTTGACAGGTTCGTACCGCATCGCTGGCACAGG
ΔfimA75::L2U1H10 donor oligonucleotide Rv	CCTGTGCCAGCGATGCGGTACGAACCTGTCAAAAAAAAAGGGCGATCATCTGACC GCCCGGCAGCGGCCAGAGCCGCTGTAGAACTGAG
ΔfimA75::L2U1H09 donor oligonucleotide Fw	CTCAGTTCTACAGCGGCTCTGGCCGCTGCCACGGCCCTAGATAGGGCCGTTTTTTT TTTGACAGGTTCGTACCGCATCGCTGGCACAGG
ΔfimA75::L2U1H09 donor oligonucleotide Rv	CCTGTGCCAGCGATGCGGTACGAACCTGTCAAAAAAAAAAACGGCCCTATCTAGGG CCGTGGCAGCGGCCAGAGCCGCTGTAGAACTGAG
ΔfimA75::L2U3H03 donor oligonucleotide Fw	CTCAGTTCTACAGCGGCTCTGGCCGCTGCCTAGCGTGACCGGCGCATCGGTCACG CTATTTGTTGAGGACAGGTTCGTACCGCATCGCTGGCACAGG
ΔfimA75::L2U3H03 donor oligonucleotide Rv	CCTGTGCCAGCGATGCGGTACGAACCTGTCCTCAACAAATAGCGTGACCGATGCG CCGGTCACGCTAGGCAGCGGCCAGAGCCGCTGTAGAACTGAG
ΔfimA75::L2U5H11 donor oligonucleotide Fw	CTCAGTTCTACAGCGGCTCTGGCCGCTGCCTAGCGTGCGAACAGCACGCTATTGTT GTATGACAGGTTCGTACCGCATCGCTGGCACAGG
ΔfimA75::L2U5H11 donor oligonucleotide Rv	CCTGTGCCAGCGATGCGGTACGAACCTGTCATACAACAATAGCGTGCTGTTCGCAC GCTAGGCAGCGGCCAGAGCCGCTGTAGAACTGAG
ΔfimA75::pheA-1 donor oligonucleotide Fw	CTCAGTTCTACAGCGGCTCTGGCCGCTGCCgacgaacaaTAAGGCCTCCCAAATCGG GGGGCCTTTTTTATTgaTaacaaaaGACAGGTTCGTACCGCATCGCTGGCACAGG
ΔfimA75::pheA-1 donor oligonucleotide Rv	CCTGTGCCAGCGATGCGGTACGAACCTGTCttttgttAtcAATAAAAAAGGCCCCCCGAT TTGGGAGGCCTTAttgttcgtcGGCAGCGGCCAGAGCCGCTGTAGAACTGAG
atpIp::L3S2P56 donor oligonucleotide Fw	tgactctaagccttaaagaaagTTTTACTCGGTACCAAATTTTCGAAAAAAGACGCTGAAAAGC GTCTTTTTTCGTTTTGGTCCtacgacacgcggcatacctcgaaggg
atpIp::L3S2P56 donor oligonucleotide Rv	cccttcgaggtatgccgcgtgtcgtaGGACCAAAACGAAAAAAGACGCTTTTCAGCGTCTTTTTT CGAAAATTTGGTACCGAGTAAAActttctttaaggcttagagtca
ΔatpIBEFHAGDG donor oligonucleotide Fw	gaacagggttagcagaaaagtcgcaattgtatgcactggaaaaatatttaaacatcaccggcttgaaaagcacaaaagc cagtctggaaacaggctggctttttttgcg
ΔatpIBEFHAGDG donor oligonucleotide Rv	cgcaaaaaaaagccagcctgtttccagactggcttttgtgcttttcaagccggtgatgtttaaatatttttccagtgcatacaatt gcgacttttctgctaaccctgttc
ΔatpB donor oligonucleotide Fw	TTTGGTGCTGGTGGTTCAGATACTGGCACCGGCTGTAATTAACAACAAAGGGTAAttt accaacactactacgttttaactgaaacaaactggagactgtcATGGAAAAC
ΔatpB donor oligonucleotide Rv	GTTTTCCATgacagtctccagtttgtttcagttaaaacgtagtagtgttggtaaaTTACCCTTTGTTGTTAATT ACAGCCGGTGCCAGTATCTGAACCACCAGCACCAAA
ΔatpB::L3S3P41 donor oligonucleotide Fw	ACCGGCTGTAATTAACAACAAAGGGTAAAAAAAAAAAAAAACACCCTAACGGGTGTTT TTTTTTTTTTGGTCTCCCtttaccaacactactacgttttaactga
ΔatpB::L3S3P41 donor oligonucleotide Rv	tcagttaaaacgtagtagtgttggtaaaGGGAGACCAAAAAAAAAAAAAAACACCCGTTAGGGTGTT TTTTTTTTTTTTTACCCTTTGTTGTTAATTACAGCCGGT

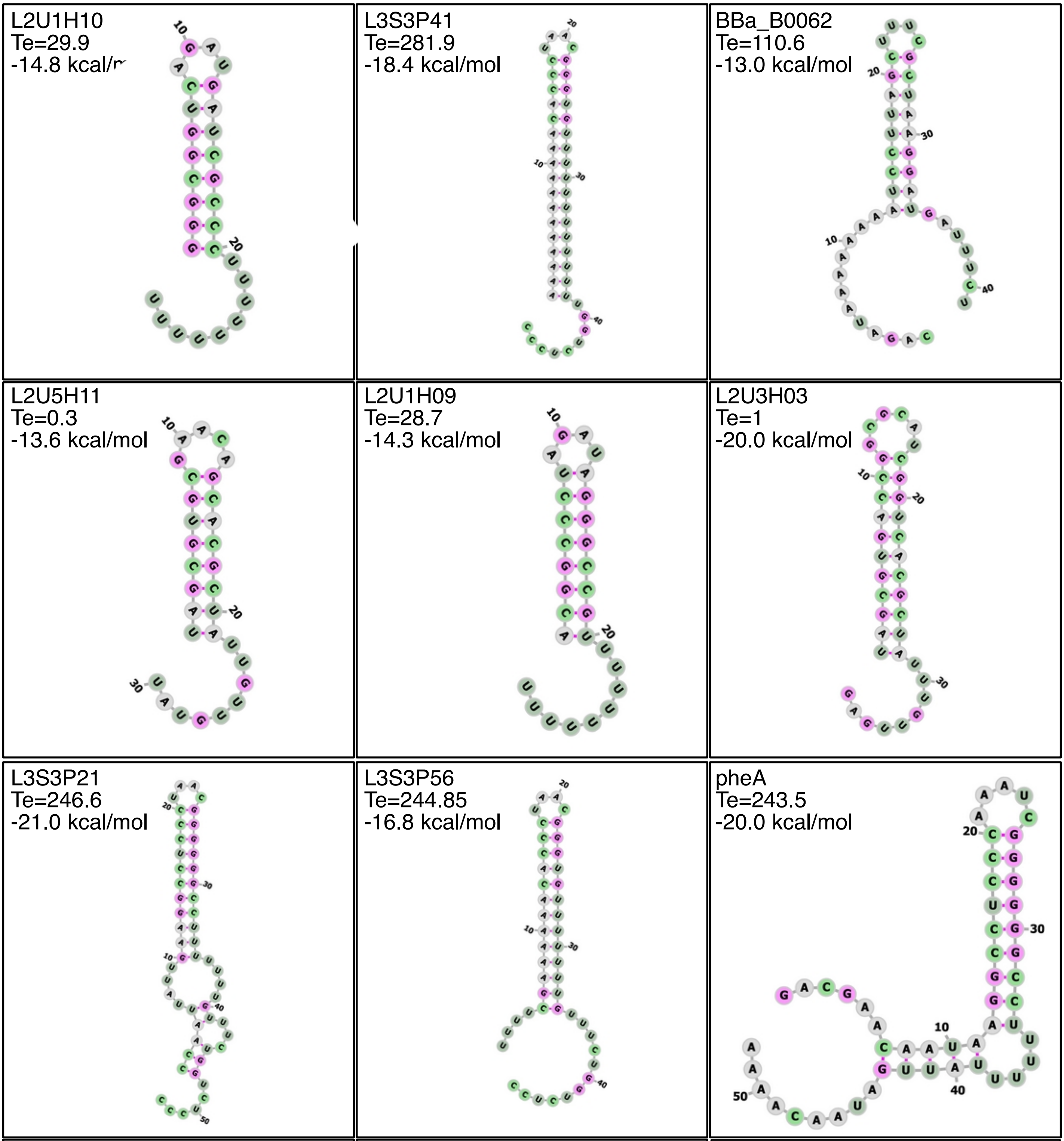

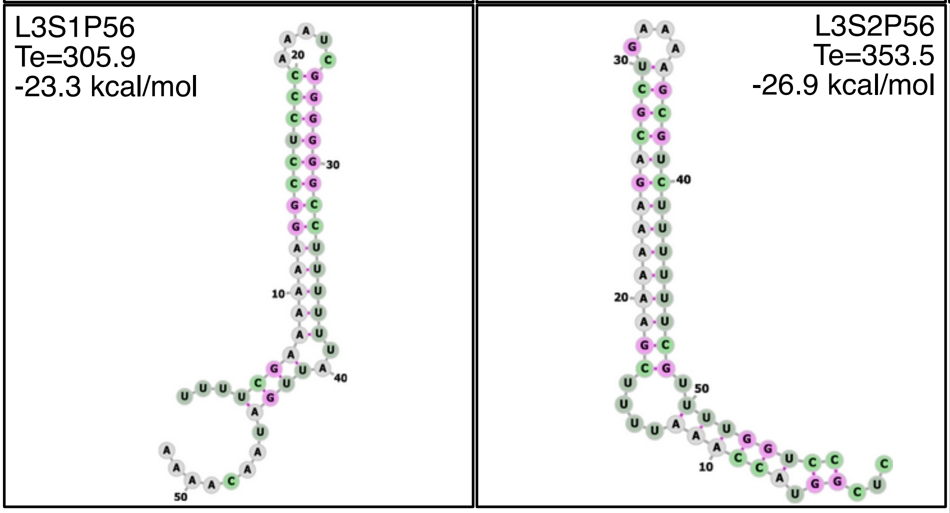

Fig. S1. Structure of terminator sequences. A selected set of terminator transcript sequences from the compilation of 582 terminator sequences characterised by Chen et al. by their termination efficiency (Te) [3].The calculated free energy of the secondary structure is shown below each transcript sequence.

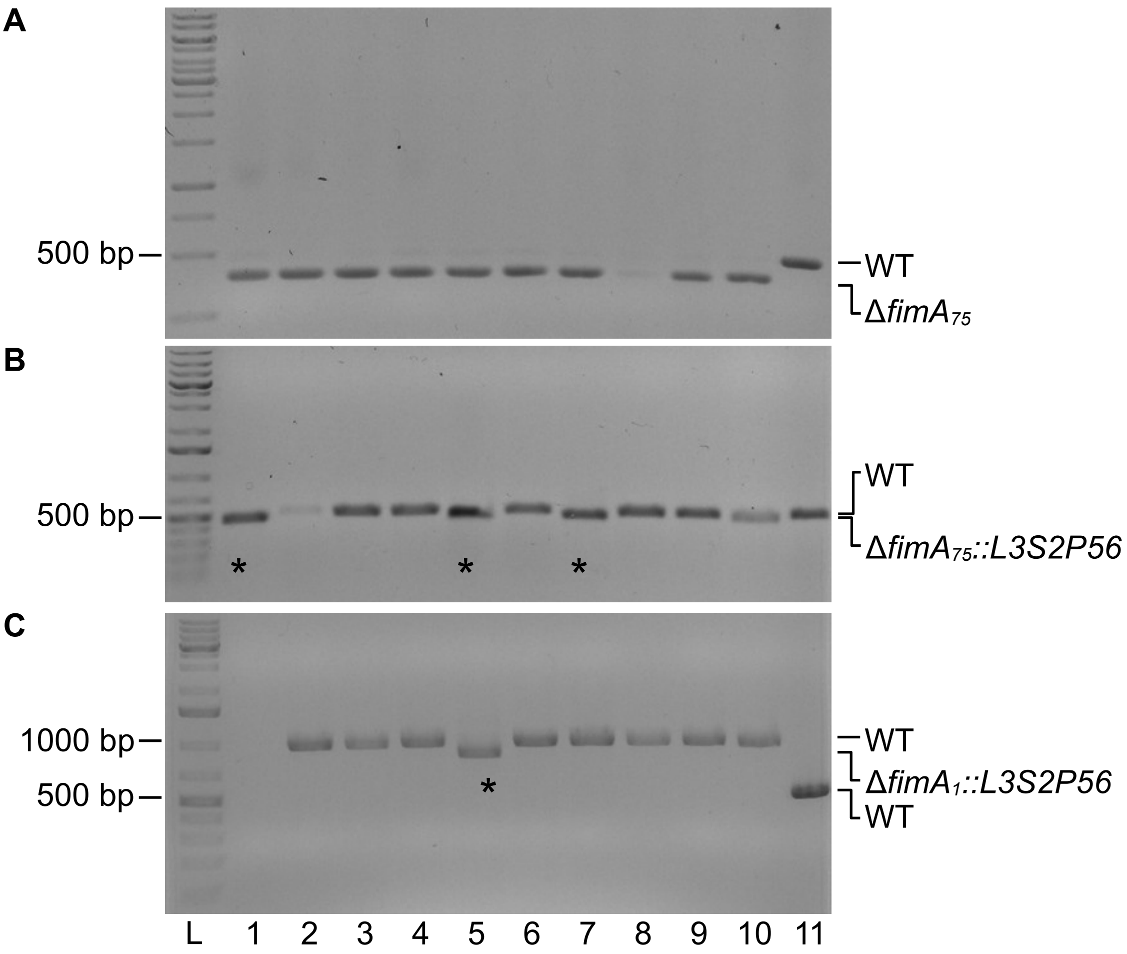

Fig. S2. Efficiency of fimA mutagenesis with Cas12a. **A** 10/10 E. coli colonies isolated after co-transformation with crRNA1 and the donor oligonucleotide carrying homology arms to introduce a 97 bp fimA deletion tested positive (lanes 1-10) for a mutation as detected by colony PCR using primers F1 and R1. **B** 3/10 E. coli colonies isolated after co-transformation with crRNA1 and the donor oligonucleotide carrying homology arms to insert a 57 bp terminator beside the 97 bp fimA deletion at position 75 of the fimA ($\Delta fimA_{75}$::L3S2P56) gene tested positive (lanes 1, 5 and 7 marked with *) as detected by colony PCR using primers F1 and R1. **C** 1/10 E. coli colonies isolated after co-transformation with crRNA1 and the donor oligonucleotide carrying homology arms to insert a 57 bp terminator sequence beside the 172 bp fimA deletion at position 1 of the fimA ($\Delta fimA_{1}$::L3S2P56) gene tested positive (lane 5, marked with *) as detected by colony PCR using primers F2 and R2. Lane 11 shows a PCR product on WT *E. coli* DNA template as detected with primers F1 and R1. Lanes labeled as L were loaded with a double-stranded DNA ladder containing fragments of different lengths in base pairs (bp).

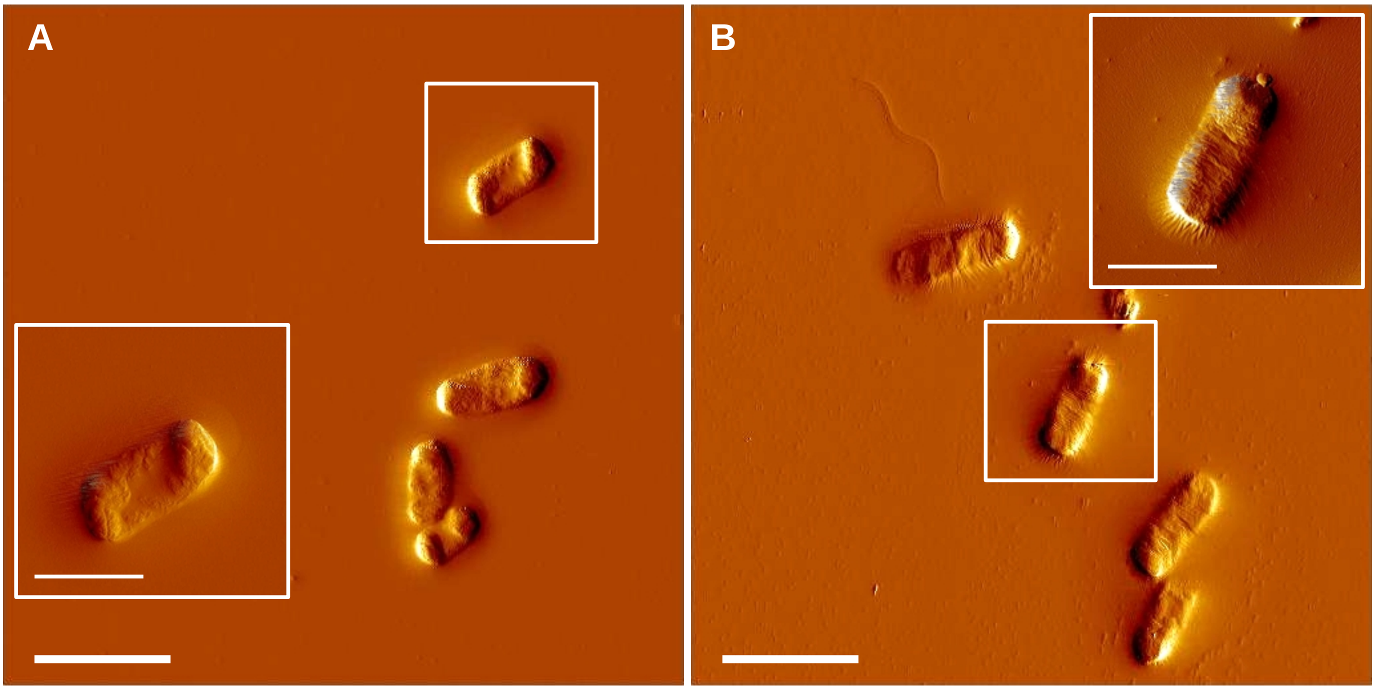

Fig. S3. Atomic force microscopy (AFM) of bacteria grown in static cultures. **A** AFM imaging of *E. coli* cells with the Δ*fimA*$_{75}$ allele carrying the empty vector pSU19. A *E. coli* cell showing no type 1 fimbriae is shown in the inset. **B** AFM imaging of the ΔfimA$_{75}$ mutant *E. coli* carrying a plasmid containing a *fimA*$^{+}$ allele for transcomplementation. A *E. coli* cell showing type 1 fimbriae is shown in the inset. Scale bars: A-B) 4 µm. A-B) insets 2 µm.

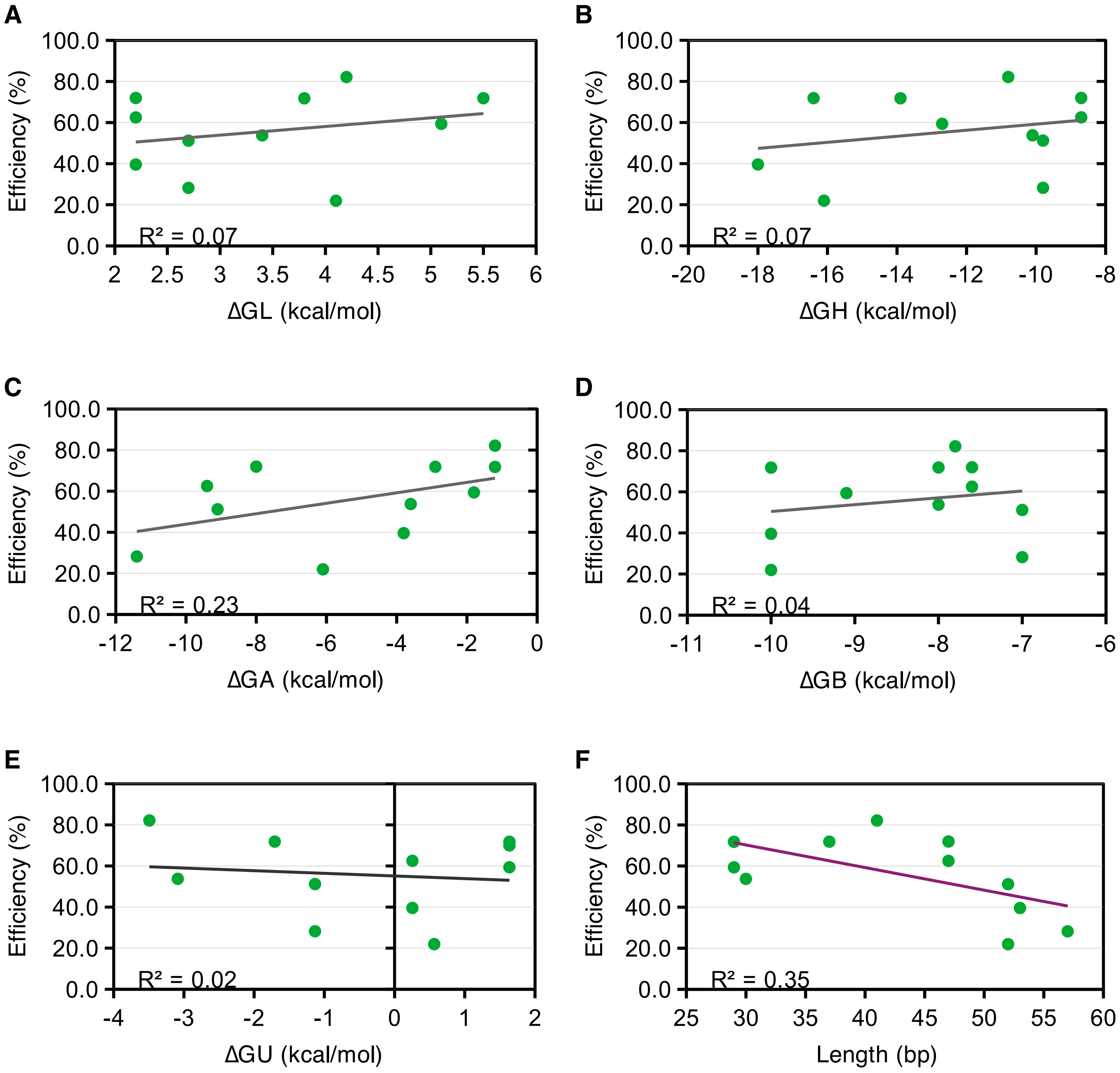

Fig. S4. Thermodynamic properties of terminator sequences. Correlation of the efficiency of mutagenesis and the following thermodynamic parameters: **A** Free energy for the closure of the hairpin loop (ΔGL). **B** Free energy of the hairpin folding (ΔGH). **C** Free energy of the extended hairpin (ΔGA). **D** Free energy of the base of the stem (ΔGB). **E** Free energy of the U-tract (ΔGU), **F** length (bp).

References

1. Guyer MS, Reed RR, Steitz JA, Low KB. Identification of a sex-factor-affinity site in E. coli as γδ. In: Cold Spring Harbor symposia on quantitative biology. Cold Spring Harbor Laboratory Press; 1981; 135–140.

2. Sansonetti PJ, Kopecko DJ, Formal SB. Involvement of a plasmid in the invasive ability of Shigella flexneri. Infection and immunity. 1982;35(3):852–860.

3. Chen Y-J, Liu P, Nielsen AA, Brophy JA, Clancy K, Peterson T, et al. Characterization of 582 natural and synthetic terminators and quantification of their design constraints. Nature methods. 2013;10:659-666.
